# *Crybb2* coding for βB2-crystallin affects sensorimotor gating and hippocampal function

**DOI:** 10.1007/s00335-013-9478-7

**Published:** 2013-10-06

**Authors:** Minxuan Sun, Sabine M. Hölter, Jens Stepan, Lillian Garrett, Just Genius, Elisabeth Kremmer, Martin Hrabě de Angelis, Wolfgang Wurst, D. Chichung Lie, Laure Bally-Cuif, Matthias Eder, Dan Rujescu, Jochen Graw

**Affiliations:** 1 Institute of Developmental Genetics, Helmholtz Center Munich – National Research Center for Environmental Health, Ingolstädter Landstrasse 1, 85764 Neuherberg, Germany; 2 Max Planck Institute of Psychiatry, 80804 Munich, Germany; 3 Divisions of Molecular and Clinical Neurobiology, Ludwig-Maximilians-University, 80336 Munich, Germany; 4 Department of Psychiatry, Ludwig-Maximilians-University, 80336 Munich, Germany; 5 Institute of Molecular Immunology, Helmholtz Center Munich – National Research Center for Environmental Health, 81377 Munich, Germany; 6 German Mouse Clinic, Institute of Experimental Genetics, Helmholtz Center Munich – National Research Center for Environmental Health, 85764 Neuherberg, Germany; 7 Chair of Experimental Genetics, Center of Life and Food Sciences, Technische Universität München, 85354 Freising-Weihenstephan, Germany; 8 Research Unit of Zebrafish Neurogenetics, Helmholtz Center Munich – National Research Center for Environmental Health, 85764 Neuherberg, Germany; 9 Present Address: Department of Psychiatry, University of Halle, 06112 Halle, Germany; 10 Present Address: Institute of Biochemistry I, Jena University Hospital, Friedrich-Schiller-University, 03641 Jena, Germany; 11 Present Address: Zebrafish Neurogenetics Group, Laboratory of Neurobiology and Development, CNRS UPR3294, Institute of Neurobiology Alfred Fessard, 91198 Gif-sur-Yvette, France; 12 Present Address: Institute of Biochemistry, Emil Fischer Center, University of Erlangen-Nürnberg, 91054 Erlangen, Germany

## Abstract

βB2-crystallin (gene symbol: *Crybb2*/*CRYBB2*) was first described as a structural protein of the ocular lens. This gene, however, is also expressed in several regions of the mammalian brain, although its function in this organ remains entirely unknown. To unravel some aspects of its function in the brain, we combined behavioral, neuroanatomical, and physiological analyses in a novel *Crybb2* mouse mutant, *O377*. Behavioral tests with male *O377* mutants revealed altered sensorimotor gating, suggesting modified neuronal functions. Since these mouse mutants also displayed reduced hippocampal size, we concentrated further investigations on the hippocampus. Free intracellular Ca^2+^ levels were increased and apoptosis was enhanced in the hippocampus of *O377* mutants. Moreover, the expression of the gene encoding calpain 3 (gene symbol *Capn3*) was elevated and the expression of genes coding for the NMDA receptor subunits was downregulated. Additionally, the number of parvalbumin-positive interneurons was decreased in the hippocampus but not in the cortex of the mutants. High-speed voltage-sensitive dye imaging demonstrated an increased translation of input-to-output neuronal activity in the dentate gyrus of this *Crybb2* mutant. These results point to an important function of βB2-crystallin in the hippocampal network. They indicate pleiotropic effects of mutations in the *Crybb2* gene, which previously had been considered to be specific to the ocular lens. Moreover, our results are the first to demonstrate that βB2-crystallin has a role in hippocampal function and behavioral phenotypes. This model can now be further explored by future experiments.

**Electronic supplementary material:**

The online version of this article (doi:10.1007/s00335-013-9478-7) contains supplementary material, which is available to authorized users.

## Introduction

For more than 100 years, crystallins have been considered structural proteins of the ocular lens, accounting for up to 90 % of the entire protein content. However, there is increasing evidence that crystallins have pleiotropic effects in different organs: α-crystallins have been associated with neurodegenerative diseases like Alzheimer’s disease, Parkinson’s disease, or Alexander’s disease (for a review see Graw 2009 and references therein). Moreover, it has been demonstrated that αA-crystallin is involved in maintaining dopaminergic neurons in the olfactory bulb (Ninkovic et al. 2010).

One of the most prominent members of the crystallins is the βB2-crystallin (gene symbol *CRYBB2* in humans and *Crybb2* in mice). The βB2-crystallin is characterized by four Greek key motifs, each encoded by its own exon. A Greek key motif consists of four adjacent antiparallel β-strands and their linking loops (Wistow 2012). The β-crystallins consist of an N- and C-terminal globular domain, each of which comprises two topologically equivalent Greek key motifs. In the native βB2-crystallin dimer, the N-terminal domain of each monomer interacts with the C-terminal domain of the other forming a βB2-crystallin homodimer (Trinkl et al. 1994). These Greek key motifs define the β/γ-crystallin superfamily and are considered to be important for the folding of these proteins, allowing their dense packaging in the lens. Mutations in the *CRYBB2*/*Crybb2* gene lead to cataracts, which are characterized by the loss of lens transparency. In humans, a frequent feature of mutations in the *CRYBB2* gene is gene conversion to a closely linked pseudogene (ψ*CRYBB2*). In mice, three mutations have been described (*Philly*, *Aey2*, and *O377*), characterized by dominant, progressive cataracts (for review see Graw 2009, and references therein).

The most recently described mutant is the O377 allele; it is characterized as an A → T substitution at the end of intron 5. The mutation forms an alternative splice site and consequently a 57-bp insertion in the mRNA, and it leads to 19 additional amino acids in the protein, just in front of the fourth Greek key motif. The predicted structure of the mutated βB2-crystallin shows an additional loop near the carboxyl terminus (Ganguly et al. 2008). Moreover, initial experiments in our laboratory with recombinant proteins demonstrated that the O377 βB2-crystallin is insoluble, making it impossible to apply common biochemical or biophysical methods to further characterize this protein (M. Sun, unpublished observation).

Besides being a structural protein in the ocular lens, there is evidence that βB2-crystallin has additional functions. *Philly* mutants suffer from reduced fertility (Duprey et al. 2007) and *Crybb*2 is upregulated in the adult regenerating retina and involved in axonal regeneration (Liedtke et al. 2007). βB2-crystallin can be phosphorylated in a cAMP-dependent manner (Kleiman et al. 1988) and is found to be phosphorylated in old lenses at eight different sites, most of which are Ser residues (Huang et al. 2011). Most interesting in the context of our experiments reported here, βB2-crystallin also has moderate ability to bind calcium, suggesting a role as calcium buffer (Jobby and Sharma 2007). This Ca^2+^-binding activity of the β/γ-crystallin superfamily was originally described in the protein S from *Myxococcus xanthus* and further validated in several members of the β/γ-crystallin superfamily, including βB2-crystallin. Most of the β/γ-crystallins have affinities in the micromolar range (4–250 μM) (Aravind et al. 2009).

Recently, we demonstrated *Crybb2* expression in different brain regions, including the hippocampus, olfactory bulb, cerebellum, and cerebral cortex (Ganguly et al. 2008), but its role in the brain is still unknown. Based on these expression sites, we hypothesized that the corresponding mouse mutant, *O377*, may display central nervous system deficits. Here we describe for the first time behavioral alterations in a *Crybb2* mouse mutant together with some molecular, cellular, and electrophysiological studies. We obtained converging evidence that the βB2-crystallin mutation interferes with the functional integrity of the hippocampus.

## Materials and methods

### Mice

Mice were kept at the Helmholtz Center Munich, Neuherberg. All animal experiments were carried out in accordance with the regulations of the German Law on Animal Protection and institutional guidelines. The *O377* mice were characterized previously on a C3H background; after 20 generations on a C3H background, homozygous mutants were crossed back to C57BL/6J for 8 generations and thereafter maintained as an intercross line (Ganguly et al. 2008). For the experiments described here, only male mice were used.

### Behavioral analysis in mice

The open field test and prepulse inhibition (PPI) of the acoustic startle reflex (ASR) were assessed according to the standardized phenotyping screens developed by the Eumorphia partners (Mandillo et al. 2008), available as EMPReSSslim protocols (see www.eumodic.org). The results were obtained by an experimenter blinded to the genotype conditions.

The open field apparatus consisted of a transparent and infrared light-permeable acrylic test arena with a smooth floor (internal measurements 45.5 × 45.5 × 39.5 cm). Illumination levels were set at ~150 lux in the corners and 200 lux in the middle of the test arena. Mice were placed individually in a corner of the arena and allowed to freely explore it for 20 min. Data were recorded and analyzed using the ActiMot system (TSE, Bad Homburg, Germany).

The ASR/PPI protocol was adapted to the specifications of our startle equipment (Med Associates Inc., St. Albans, VT, USA). Background noise [no stimulus (NS)] was 65 dB and trial types for ASR included seven different stimulus intensities (NS, 70, 80, 85, 90, 100, 110, and 120 dB). Trial types for PPI included four different prepulse intensities (67, 69, 73, and 81 dB), with each prepulse preceding the startle pulse (110 dB) by a 50-ms interstimulus interval. Each stimulus type was assessed ten times, and trial types were arranged in blocks of ten in random order.

Social discrimination was assessed as previously described (Feil et al. 2009). The procedure consisted of two 4-min exposures of stimulus animals (ovariectomized 129Sv females) to the test animal in a fresh cage to which the test animal had been moved 2 h prior to testing. During the first exposure, one stimulus animal was exposed to the test animal. After a retention interval of 2 h, this stimulus animal was re-exposed to the test animal together with an additional, previously not presented stimulus animal. The duration of investigatory behavior of the test animal toward the stimulus animals was recorded by a trained observer with a hand-held computer. A social recognition index was calculated as time spent investigating the unfamiliar stimulus mouse/time spent investigating both the familiar and unfamiliar stimulus mouse.

Spontaneous alternations were assessed using the Y-maze, which was made of opaque light gray PVC and had three identical arms (30 × 5 × 15 cm) placed at 120° from each other; illumination in the center of the maze was 100 lux (Wall et al. 2003). Each mouse was placed at the end of one arm and allowed to move freely through the maze during a 5-min session. Spontaneous alternations (defined as consecutive entries into all three arms without repetitions in overlapping triplet sets) were scored. The total number of arm entries was collected over the 5 min. Spontaneous alternation performance percentage is defined as the ratio of actual (total) alternations to possible alternations (total number of triplets) × 100. When placed in the Y-maze, normal mice prefer to explore the least recently visited arm and thus they tend to alternate visits among the three arms. To explore the three arms successively, the mouse must maintain an ongoing record of the most recently visited arm and continuously update the record. Therefore, alternation behavior is a measure of spatial working memory.

**Fig. 1:**
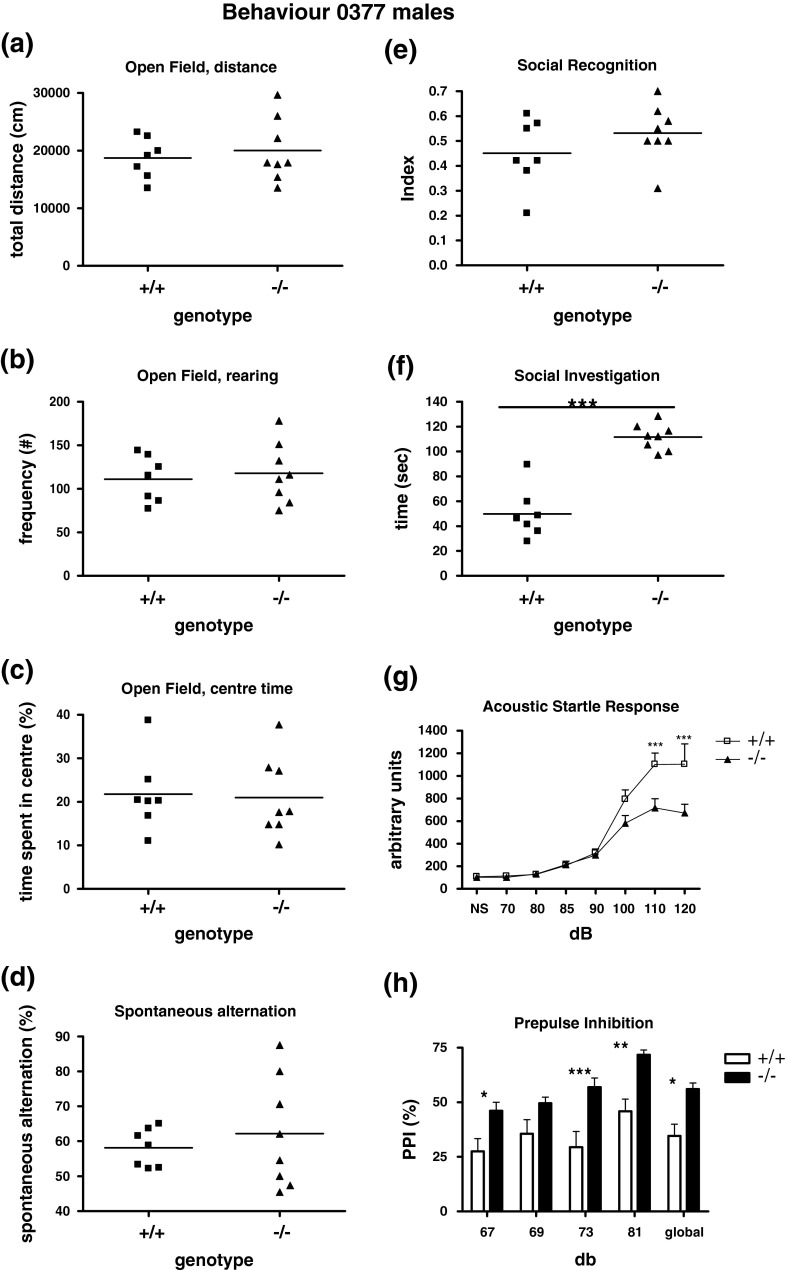
Behavioral analysis of male *O377* mutant mice and wild-type littermate control mice (*n* = 5–10). **a**–**c** Spontaneous exploratory behavior in the open field. No differences in horizontal (**a**) or vertical (**b**) locomotor activity. **d** No difference in working memory as indicated by spontaneous alternation in the Y-maze. **e**, **f** Social investigation of the stimulus animal during the sample phase (first exposure) of the social discrimination test. **f** *O377* mutants spent significantly more time in social investigation than control males. **g** There were significant differences in the acoustic startle curves of *O377* mutants in comparison to littermate controls. **h** Increased PPI in *O377* mutants. *p < 0.05; **p < 0.01; ***p < 0.001; ^+/+^ versus ^−/−^

The age of the mice was 9 weeks during the Open Field test, 11 weeks at the ASR/PPI measurement, 13–16 weeks during the social discrimination test, and 26 weeks during the Y-maze test. Data are reported as mean ± standard error of the mean (SEM) in bar or line graphs, or are individually shown in scatterplots with a horizontal line indicating the mean. Data were statistically analyzed by two-way analysis of variance (ANOVA) (Fig. 1g, h) or by unpaired Student’s *t* test (Fig. 1a–f). The chosen level of significance was *p* < 0.05.

### Morphological and histological analysis

Brains from adult mice (transcardially perfused with 4 % paraformaldehyde in PBS) were transferred to a 30 % sucrose solution for 48 h. Coronal brain sections 40 μm thick were made using a sliding microtome. Cresyl violet staining was used to analyze the differences in hippocampal morphology between mutants and wild-type mice. The volumes of dentate gyrus (DG) and hippocampus were measured under a microscope with Stereo Investigator software (MBF Bioscience, Williston, VT, USA). For neurogenesis assessment, 3-month-old *O377* mutants and C57BL6 wild-type mice were injected with bromodeoxyuridine (BrdU; 50 mg/kg body weight i.p.) once a day for five continuous days. Then the animals were perfused 1 month after the first injection.

### Hippocampal volume measurement

1- and 3-month-old wild-type and *O377* male mice were sacrificed and 40-μm-thick free-floating sections were prepared as described previously (Feil et al. 2009). Every 12th section was selected and stained with Nissl staining. The volumes of the regions of interest (ROIs) [DG, *cornu ammonis* (CA), and hippocampus] were measured as previously described (Boldrini et al. 2009).

### Manufacturing of monoclonal antibody against mouse βB2-crystallin

A new monoclonal antibody against βB2-crystallin was generated by cloning the full sequence of *Crybb2* into the pETM11 vector. This construct was then transfected in *Escherichia coli* BL21/DE3, and after IPTG (isopropyl-1-thiogalactopyranoside) induction, a soluble lysozyme extract was purified via Ni resin (GE Healthcare, Piscataway, NJ, USA). Shortly thereafter, the 6His fusion protein was eluted from the column with elution buffer (50 mM Tris, 500 mM NaCl, 250 mM imidazol, and 1 mM TCEP, pH 7.4). Salts were removed using a Sephadex G-25 column (NAP-25 column, GE Healthcare). The antigen was suspended in PBS and then used for immunization. Fifty micrograms of the purified N-His-fusion protein (βB2-crystallin) was injected intraperitoneally (i.p.) and subcutaneously (s.c.) into LOU/C rats using incomplete Freund’s adjuvant supplemented with 5 nmol CpG 2006 (TIB MOLBIOL, Berlin, Germany). After 6 weeks a final boost with 50 μg of βB2-crystallin and CpG 2006 was given i.p. and s.c. 3 days before fusion. Fusion of the myeloma cell line P3X63-Ag8.653 with rat immune spleen cells was performed according to standard procedures. Hybridoma supernatants were tested in a solid-phase immunoassay with βB2-crystallin or an irrelevant N-His fusion protein coated to ELISA plates. Antibodies from tissue culture supernatant bound to βB2-crystallin were detected with HRP (horseradish peroxidase)-conjugated monoclonal antibodies against the rat IgG isotypes [TIB173 IgG2a, TIB174 IgG2b, and TIB170 IgG1, all from ATCC (Manassas, VA, USA), and R-2c IgG2c (Ascenion, Munich, Germany)], thus avoiding monoclonal antibodies of IgM class. Hypothalamic–pituitary–gonadal (HPG) activity was visualized with ready-to-use TMB (tetramethylbenzidine) (1-step™ Ultra TMB-ELISA, Thermo Fisher Scientific, Waltham, MA, USA). Monoclonal antibodies that reacted specifically with βB2-crystallin were further analyzed; in this study, we used the antibody BB2-4C4 (rat IgG2a).

### In situ hybridization

Mouse brain tissue was frozen in isopentane at −30 °C and stored at −80 °C. cRNA probes were generated from cloned inserts into the pCRII-TOPO vector (Invitrogen, Carlsbad, CA, USA). Cryosections were fixed in 4 % paraformaldehyde, and in situ hybridization was processed as described previously (Haupt et al. 2007).

### RNA isolation, cDNA production, reverse-transcription PCR, and quantitative real-time PCR

Total RNA was isolated using the RNA-Bee (AMS Biotechnology, Abingdon, UK). Isolated RNA was treated with DNase (Promega, Madison, WI, USA) according to the manufacturer’s protocol. cDNA was synthesized using the T-Primed First-strand Kit (Amersham Biosciences, GE Healthcare, Piscataway, NJ, USA) for reverse-transcription PCR and quantitative real-time PCR. Reverse-transcription PCR was performed on a PCR thermal cycler (MJ Research, Bio-Rad, Hercules, CA, USA) using the *Taq* DNA polymerase (Invitrogen); the PCR mixture was made following the manufacturer’s instructions. Primers used for PCR were as follows: *Crybb2* forward: 5′-AAGCTAGCATGGCCTCAGACCACCAG-3′, reverse: 5′-AAGGATCCGCTGGAGGGGTGGAAG-3′; *Gapdh* forward: 5′-ACGCTAGCATGGTGAAGGTCGGTGT-3′, reverse: 5′-AAGGATCCCTCCTTGGAGGCCATGT-3′.

Quantitative real-time PCR was performed on a step one device (Applied Biosystems, Life Technologies, Carlsbad, CA, USA). EvaGreen^®^ qPCR Master Mix (Solis BioDyne, Tartu, Estonia) was used according to the manufacturer’s protocol. In quantitative real-time PCR, *Tuba1a* was used as a control; primers for real-time PCR were as follows: *Capn3* forward: CTATGAATCATCACCATGCGCTA, reverse: CATACATGGTAAGCTGCAGCCA; *Grin1* forward: TTAAGGTGAACAGCGAGGAG, reverse: CAGGTTGGCAGTGTAGGAAG; *Grin2a* forward: TCTCCTCACAGACTTTCATCCCC, reverse: GTGACCAAGGAGAAGACATGCC; *Grin2b* forward: TGGGGGCTCATCTATGATAATGG, reverse: GCGGATCTTGTTCACGAAGTC: *Grin2c* forward: CTCTGTGCCTTTTGTGGAGACC, reverse: GGTTGTAGCTGACAGGGCTGAA; *Tuba1a* forward: CCAGATGCCAAGTGACAAGA, reverse: GTGGGTTCCAGGTCTACGAA; *POLR2A* forward: 5′-AGACCGGCTATAAGGTGGAA-3′, reverse: 3′-CTGCCGGTTGAAGATAACAA-5′; *CRYBB2* forward: 5′-CATCAAAGTGGACAGCCAAG-3′, reverse: 3′-GAAGCTGGGTACATCGTCATG-5′.

### Immunohistochemistry and co-labeling analysis

Sections were fixed and processed for immunohistochemistry as previously described (Ehm et al. 2010). For detection of βB2-crystallin, we used a novel monoclonal antibody (BB2-4C4; rat IgG2a) whose manufacture was described above. Further primary antibodies used in this study included rabbit anti-parvalbumin (1:1,000; Swant, Bellinzona, Switzerland), rabbit anti-calbindin (1:1,000, Swant), rabbit anti-Ki67 (1:200; Abcam, Cambridge, MA, USA), rabbit anti-cleaved caspase-3 (1:100; BD Pharmingen, San Diego, CA, USA), rabbit anti-somatostatin (1:500; Enzo Life Sciences, Farmingdale, NY, USA). Secondary antibodies (donkey anti-rabbit-Cy3 and donkey anti-rat-alex488) were obtained from Jackson ImmunoResearch Laboratories (West Grove, PA, USA) and Invitrogen, respectively. DAPI (10 mg/ml) (Sigma-Aldrich, St. Louis, MO, USA) was used as a control. Images were obtained using an Olympus FluoView 1000 or a Leica SP5 confocal microscope. Confocal images and *Z*-stacks were taken on a FluoView 1000. Quantification of βB2-crystallin/parvalbumin, βB2-crystallin/calretinin, and βB2-crystallin/somatostatin was performed on the prefrontal cortex, ventral DG, and ventral CA. In every group, over 50 cells were randomly selected from every sixth 40-μm section of the brain sections.

### Unbiased stereological cell counting

Cells expressing parvalbumin, calretinin, somatostatin, and cleaved caspase-3 were counted using the Stereo Investigator 5.05.4 software on every sixth serial 40-μm coronal free-floating section. All the positive cells in the prefrontal cortex, ventral DG, and ventral CA were counted. At the same time, the volume of the counted regions was also measured using the Stereo Investigator 5.05.4 software. DAPI staining was used as counterstain and to calculate cell density (cells/mm^3^) for each region.

### Calcium concentration measurement

Hippocampal neurons were isolated from the brains of E17.5–E18.5 wild-type and *O377* embryos. Hippocampi were dissected free of meninges, cut into small pieces, and digested with trypsin, and the cells were collected. The calcium measurement procedure was based on previous descriptions (Grynkiewicz et al. 1985; Díaz-Hernández et al. 2002). Hippocampal neurons were washed twice in HBSS + (137 mM NaCl, 5.8 mM KCl, 10 mM HEPES, supplemented with 5 mM glucose, 2.5 mM CaCl_2_, 10 μM glycine, and 1 mM MgCl_2_) by resuspension and subsequent centrifugation [1,000 rpm, 10 min at room temperature (RT)]. After cell counting and a viability check in a Neubauer chamber using Trypan blue dye exclusion (0.2 %), the cell concentration was adjusted to 1 × 10^6^ cells/ml. Then the cells were incubated with 2.5 μM of the calcium dye Fura-PE3/AM for 30 min at 37 °C in a humidified 5 % and CO_2_ 95 %. Afterward, the extracellular dye was removed by HBSS+ rinsing and centrifugation (1,000 rpm, 10 min at RT). Before measurements, dye desertification was allowed for a further 30 min. For measurements, 2 ml of the hippocampal neuron suspension at a concentration of 0.5 × 10^6^ cells/ml was transferred to a cuvette equipped with a magnetic stirrer and a thermostatted waterjacket kept at 37 °C.

Fluorescence ratios (*F*_380nm_/*F*_340nm_) proportional to Ca^2+^ concentration were monitored in a LS50B spectrofluorimeter (PerkinElmer, Waltham, MA, USA). NMDA (50 μM) was used to open the NMDA receptor to influx calcium ions from the environment to the cell cytoplasm after a stable Ca^2+^ level was obtained. Then, 10 μM thapsigargin was used to allow an influx of calcium into the cytosol from the endoplasmic reticulum. The calibration was performed by adding 10 μM ionomycin (*R*_max_) and 5 mM EGTA (*R*_min_). Ca^2+^ concentrations were calculated from the raw data using the Grynkiewicz formula (Grynkiewicz et al. 1985): [Ca^2+^]_I_ = *k*_*d*_ (*F*_min, 380nm_/*F*_max, 380nm_)[(*R* − *R*_min_)/(*R*_max_ − *R*)], where *k*_*d*_ = 146 nmol/l.

### Electrophysiology

#### Recording of mIPSCs

Using standard procedures, whole-cell voltage-clamp recordings (−70 mV holding potential) of miniature inhibitory postsynaptic currents (mIPSCs) in DG granule cells were taken at RT (23–25 °C) in 400-μm-thick coronal brain slices. The extracellular solution, which was continuously bubbled with carbogen gas, consisted of (in mM) 125 NaCl, 2.5 KCl, 25 NaHCO_3_, 1.25 NaH_2_PO_4_, 2 CaCl_2_, 1 MgCl_2_, 25 glucose, 0.005 NBQX, 0.05 AP5, and 0.001 TTX. The patch-pipette solution contained (in mM) 100 Cs-methane sulfonate, 60 CsCl, 5 QX-314, 10 Hepes, 0.2 EGTA, 1 MgCl_2_, 1 Mg-ATP, and 0.3 Na_3_GTP. Analysis of mIPSCs was done using the Mini Analysis Program (Synaptosoft, Decatur, GA, USA).

#### Voltage-sensitive dye imaging (VSDI)

Preparation and staining of brain slices, VSDI, and data analysis were performed as previously described (von Wolff et al. 2011) with the following modifications. We used a custom-made monopolar tungsten electrode (Teflon-insulated to the tip of 50 μm in diameter) for electrical stimulation of the perforant path. This electrode allowed for very precise placement into the neuronal tissue and did not interfere with VSDI (e.g., by producing irritating shadows as is seen with other electrodes). A highly localized electrical stimulation was achieved by positioning the indifferent electrode far away from the slice in the recording chamber. The electrode was placed on the visually identified perforant path near its entry zone to the DG (Fig. 6a). ROI1 and ROI2 were created by the polygon-drawing function of the MiCAM02 software.

## Results

### Behavioral studies in mice

Because of the observed *Crybb2* expression in different brain regions, we first investigated the behavior of homozygous male *O377* mutants. Analysis of spontaneous activity in a novel environment, as measured by the Open Field test, did not reveal any genotype effects in forward (total distance) or vertical (rearing) exploratory activity in *O377* mice (Fig. 1a, b), nor in anxiety-related behavior as measured by the time spent in the aversive center of the open field (Fig. 1c). Assessment of working memory by analysis of spontaneous alternations in the Y-maze also did not reveal any genotype effect in *O377* mice (Fig. 1d). When tested for social investigation, male *O377* mutants did not reveal any deficit in social recognition (Fig. 1e) but spent significantly more time in social investigation than control littermates (Fig. 1f; Student’s *t* test, *t*_(13)_ = 7.601, *p* < 0.0001). A simple test for olfactory function (on C3H background), in which soiled bedding from a cage of unfamiliar male mice is presented in a tube together with another tube containing clean, fresh bedding, indicated that homozygous male *O377* mutants can smell a social odor as well as the control mice (data not shown). This result, with the lack of a recognition deficit, suggests that the observed increase in social investigation is unlikely to be due to an olfactory deficit. Moreover, we performed social discrimination experiments to assess olfaction-based social memory and tested spontaneous alternation in the Y-maze to assess working memory and could not detect any deficits (data not shown).

The most prominent behavioral phenotype was observed in the PPI of the ASR. Assessment of the ASR revealed a significant reduction of the startle response of the *O377* mice compared to the controls at the highest sound intensities, 110 and 120 dB [Fig. 1g, two-way ANOVA for the factors genotype and startle stimulus intensity (dB), interaction genotype × dB: *F*_(1,7)_ = 5.703, *p* < 0.0001; post hoc test for 70–100 dB was not significant, post hoc tests for 110 and 120 dB, *p* < 0.001]. Moreover, PPI measurements revealed a clearly increased PPI in comparison to littermate controls in *O377* mutants [Fig. 1h; two-way ANOVA for the factors genotype and prepulse stimulus intensity (dB), genotype effect: *F*_(1,52)_ = 13.43, *p* < 0.01].

### Hippocampal volume was reduced in *O377* mouse mutants

During routine preparation of brain sections, we repeatedly observed smaller hippocampi in *O377* compared to wild-type mice (for an overview, see the serial section in Supplementary Fig. 1). To find out whether the smaller size might be associated with *Crybb2* expression, we checked the gene expression using RT-PCR and found *Crybb2* expressed from at least the embryonic stages to adult (we checked from E14 to 3 months after birth; Supplementary Fig. 2). For a systematic comparison of the hippocampal volume, brain sections were stained with Cresyl violet and the volumes of the CA area, DG, and whole hippocampus were determined in the brains of wild-type and *O377* mice at 1 and 3 months. The entire hippocampal volume was significantly decreased by 18.4 % and 19.2 % in 1- and 3-month-old *O377* mutants compared to wild-type (for details see Supplementary Fig. 3; Supplementary Table 1).

### Reduced size of the hippocampus of βB2-crystallin mutant mice runs parallel with increased apoptosis

A reason for a reduced size of the hippocampus might be an altered balance between neurogenesis and cell death. To determine whether neurogenesis was affected by impaired βB2-crystallin function, we first stained the brain sections with the proliferation marker Ki67. The cell density of Ki67 was not significantly different at the DG of 1- and 3-month-old wild-type and *O377* mice (Supplementary Fig. 4). To confirm this result, we applied the thymidine analog bromodeoxyuridine (BrdU) to 3-month-old mice and quantified after a 4-week chase the number of labeled cells incorporated into the hippocampus. Again, there was no statistically significant difference between the hippocampus of wild-type and *O377* mice (Supplementary Fig. 5), suggesting the normal generation and survival of new cells during adulthood. Next, to assess neuronal maturation, in the same paradigm we monitored the proportion of BrdU-positive cells expressing the postmitotic neuroblast marker Doublecortin (DCX) and the neuronal marker NeuN (Neuronal Nuclei). The density of BrdU;DCX;NeuN triple-labeled cells in the hippocampus was comparable in wild-type and *O377* mutant mice (Supplementary Fig. 6). We conclude that neurogenesis parameters are not affected by impaired functioning of βB2-crystallin.

**Fig. 2:**
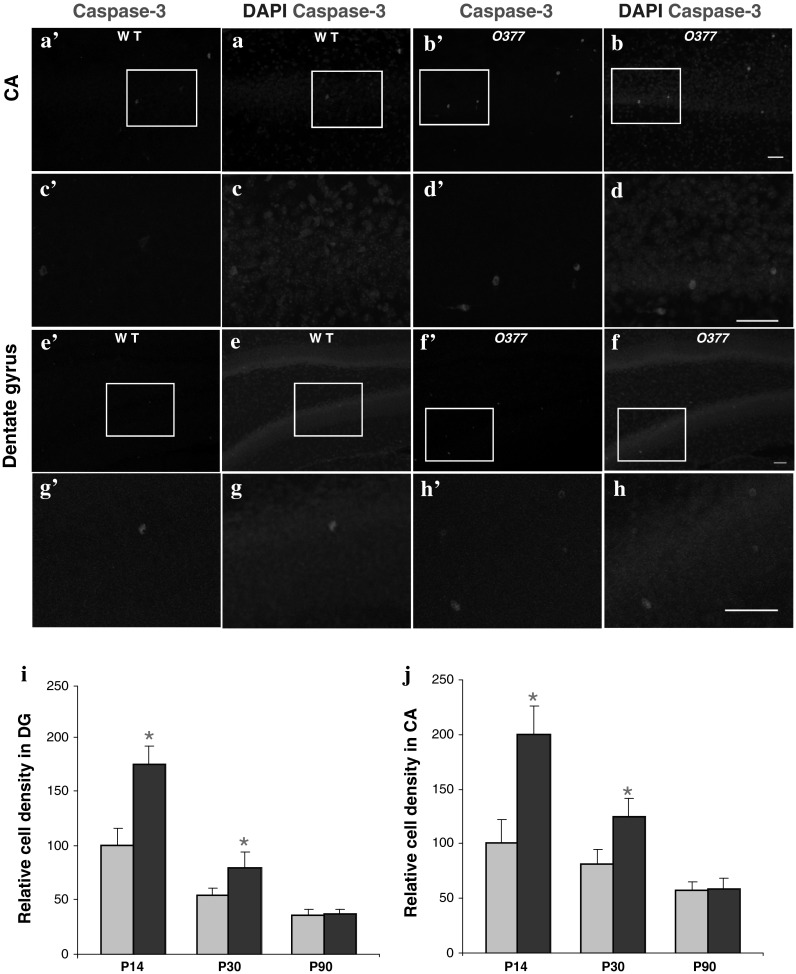
Apoptotic cell death is increased in young *O377* mutants. **a**–**h** Confocal images of anti-caspase-3 staining (*red*) in 2-week-old wild-type (WT, *left panel*) and *O377* mice (*right panel*) in the ventral CA (*upper panel*, **a**–**d**) and in the DG (*lower panel*, **e**–**h**). DAPI staining is given in *blue*. The boxed area is given below in a higher magnification. In **i**, **j**, the values are normalized to wild-type mice at the age of 3 months and given in % (T = 100 %). **i** Density of caspase-3-positive cells in WT and *O377* mutants in the ventral DG (WT = 100 %). **j** Density of caspase-3-positive cells in wild-type and *O377* mutants in the ventral CA (WT = 100 %). *p ≤ 0.05, Student’s *t* test, *n* ≥ 4. *Scale bar* 40 μm; *error bar* SEM

Next, we investigated apoptosis using immunohistochemistry with an antibody against cleaved caspase-3 (Fig. 2). In 2-week-old mice, the density of apoptotic cells was found to be increased by 75.2 % in the ventral DG of *O377* mutants in comparison to the wild-type mice; in the ventral CA of the *O377* mutants, it was twice that of the wild-type animals. Interestingly, this difference was smaller at 1 month and disappeared at 3 months of age. Immunofluorescence for double labeling of parvalbumin and cleaved caspase-3 was also performed; however, no double-labeled cells were detected. Together, this suggested that the reduced size of the hippocampus (and concomitantly, also the number of parvalbumin-positive neurons) of βB2-crystallin mutants might be an indirect effect of a wave of cell death prominent between 2 and 4 weeks after birth.

### Altered calcium homeostasis associated with impaired βB2-crystallin

**Fig. 3:**
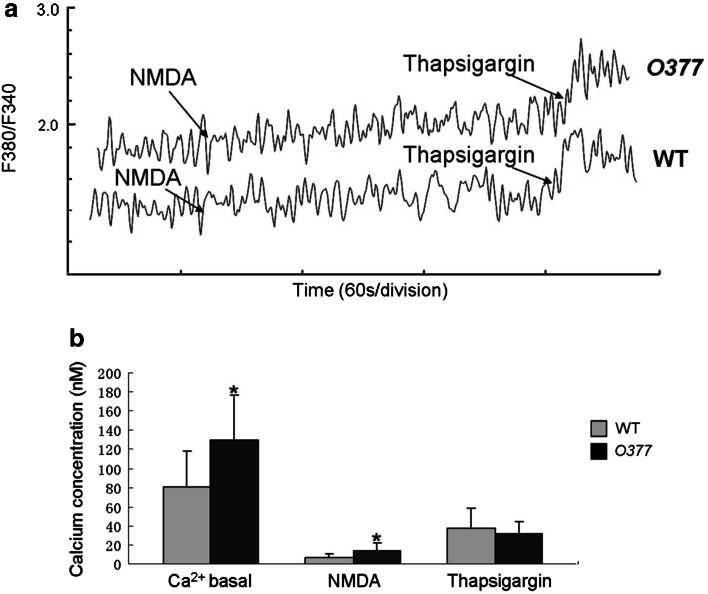
Calcium concentration is increased in embryonic *O377* hippocampal neurons. **a** Representative calcium recording in hippocampal neurons of wild-type and *O377* mutant embryos at E17.5–E18.5. The fluorescence ratio (*F*_380_/*F*_340_) is proportional to the Ca^2+^ concentration. **b** Basal calcium concentration and calcium concentration after stimulation by NMDA are significantly higher in *O377* embryonic hippocampal neurons compared to the wild type; there is no statistically significant difference in the calcium release after thapsigargin treatment. For each measurement, six to eight embryos were used; the number of measurements was *n* = 10 for the wild types and *n* = 12 for the mutants. *p ≤ 0.05, Student’s *t* test. *Error bar* SEM

Since it was suggested that βB2-crystallin has Ca^2+^ binding ability (Jobby and Sharma 2007), we investigated relative Ca^2+^ concentrations in hippocampal cells of mutant mice by calcium spectrometry. The calcium-sensitive dye was easily bleached in postnatal hippocampal neurons, rendering it difficult to obtain reliable data (J. Genius; unpublished observation). Since *Crybb2* is also expressed at embryonic stages in the hippocampus (Supplementary Fig. 2), we chose to measure Ca^2+^ concentration in the late embryonic hippocampus (E17.5–E18.5), expecting similar effects of the mutated βB2-crystallin as at postnatal stages. Basal Ca^2+^ concentration in *O377* mutants was significantly increased (129.6 ± 13.5 nM) compared to the wild-type (81.0 ± 11.6 nM). After stimulation with NMDA, the increase in calcium concentration of *O377* hippocampal neurons was also significantly higher (13.6 ± 2.5 nM) than in the wild-type (7.2 ± 1.2 nM). However, after stimulation of *O377* hippocampal neurons by thapsigargin (depleting Ca^2+^ stores), the Ca^2+^ concentration was not significantly altered compared to the wild-type (Fig. 3). This indicates that the mutation in *Crybb2* leads to an increase in basal and NMDA-receptor–mediated calcium concentration in *O377* hippocampal neurons.

**Fig. 4:**
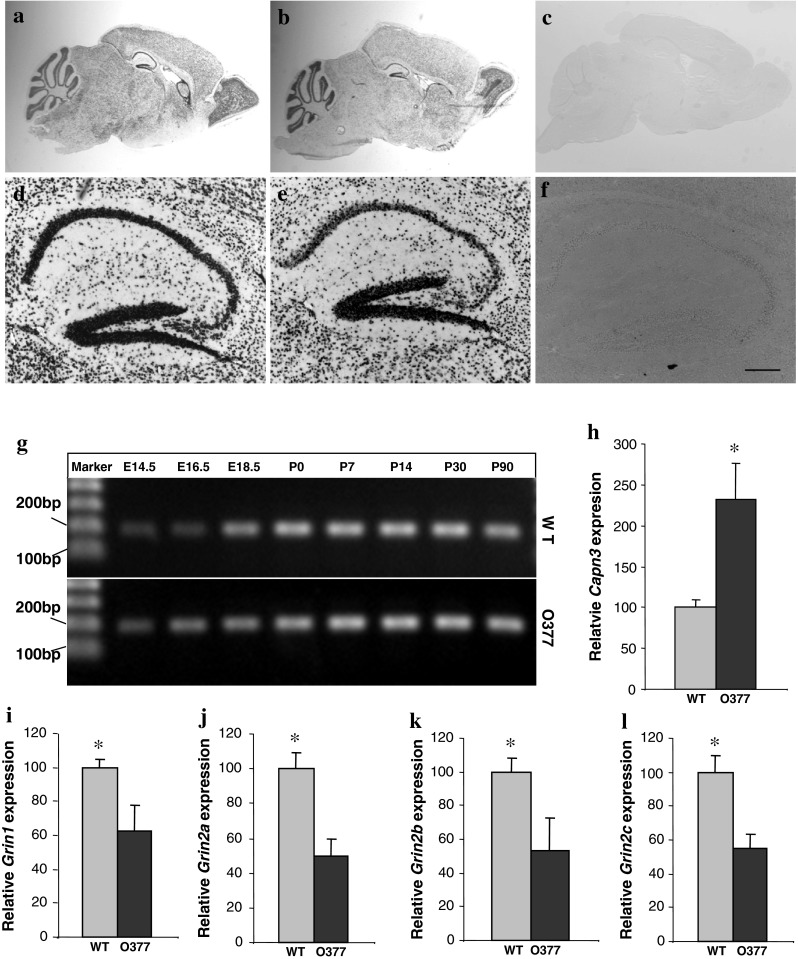
Altered expression levels of *Capn3* and *Grin1*-*Grin2C* at P14. **a**–**c** *Capn3* was detected to be expressed in the cerebellum, hippocampus, rostral migratory stream, and olfactory bulb of wild-type (**a**) and *O377* mutant mice (**b**); no staining was ever observed with the sense control (**c**). **d**–**f** Close-up of the *Capn3* expression in the hippocampus (**d** wild-type; **e** *O377*; **f** sense control). **g** *Capn3* was detected by RT-PCR in the hippocampus at different developmental stages (E14.5–E18.5) and postnatally (P0–P30) in wild-type and *O377* mice. **h**–**l** Normalized expression ratios of *Capn3* (**h**), *Grin1* (**i**), *Grin2A* (**j**), *Grin2B* (**k**), and *Grin2C* (**l**) in wild-type and *O377* mutant mice determined by real-time PCR at P14. The relative gene expression levels were calculated by the ratio of the mRNA level of the gene of interest versus the expression level of the mRNA of a housekeeping gene, *Tuba1a*, according to the 2^−ΔΔCt^ method (WT = 100 %).*p ≤ 0.05, Student’s *t* test, *n* ≥ 4. *Error bar* SEM

Previous work demonstrated the upregulation of calpain 3 (gene symbol *Capn3*) expression in the brain of adult *O377* mutants (Ganguly et al. 2008). Calpains are calcium-dependent proteases and highly associated with apoptosis (Raynaud and Marcilhac 2006). Given the results above that point to early changes in calcium homeostasis and neuronal death in βB2-crystallin mutants, we analyzed the expression of *Capn3* in the brain of wild-type and *O377* mutant mice at the age of 1 and 3 months. *Capn3* was found to be widely expressed in the brains of both genotypes. We observed positive staining in Purkinje cells and granular cells of the cerebellum, in all layers of the cerebral cortex, in the CA1, CA2, CA3, and DG regions of the hippocampus, and in the glomerular layer, mitral layer, and granule cells of the olfactory bulb pointing to an overlapping expression pattern of *Capn3* and *Crybb2* (Fig. 4a–g). We also detected *Capn3* expression in the hippocampus from E14.5 onward; it reached its maximum level at birth and remained constant at least up to 3 months (Fig. 4h). It might be important to mention that the *Capn3* expression pattern in the hippocampus is almost identical to the *Crybb2* expression pattern in the hippocampus, as described previously (Ganguly et al. 2008).

Since calpain expression has been discussed as a cause of the downregulation of NMDA receptor levels (for review see Wu and Lynch 2006), we tested this hypothesis by quantitative PCR of the corresponding genes. Indeed, we found that the expression of the NMDA receptor genes (*Grin1*, *Grin2a*, *Grin2b*, and *Grin2c*) was significantly decreased at P14 in the hippocampus of *O377* mutant mice compared to wild-type controls (between 37.4 and 50 %, Fig. 4i–l).

### Age-dependent loss of parvalbumin-positive neurons in *O377* mutants

To better understand the role of βB2-crystallin in the hippocampus, we analyzed βB2-crystallin expression using immunohistochemistry in 1- and 3-month-old wild-type mice. For comparison, we also determined βB2-crystallin expression in the prefrontal cortex. In these structures, interneurons of various chemical subclasses are present, notably expressing markers such as parvalbumin, calretinin or somatostatin (Kubota et al. 2011). We detected βB2-crystallin in the vast majority of parvalbumin-positive cells in the prefrontal cortex (96.7 ± 1.5 % of parvalbumin-positive cells expressed βB2-crystallin), CA1 (94.9 ± 2.0 %), and the DG (97.8 ± 2.6 %). βB2-crystallin expression was also detected in a subset of calretinin-positive cells in the prefrontal cortex (75 ± 2.6 %), CA (69.6 ± 5.9 %), and the DG (46.3 ± 5.2 %), and in most somatostatin-positive cells in the prefrontal cortex (90.5 ± 3.1 %), CA (92 ± 2.8 %), and the DG (92.6 ± 3.0 %) (Supplementary Figs. 7, 8, 9).

**Fig. 5:**
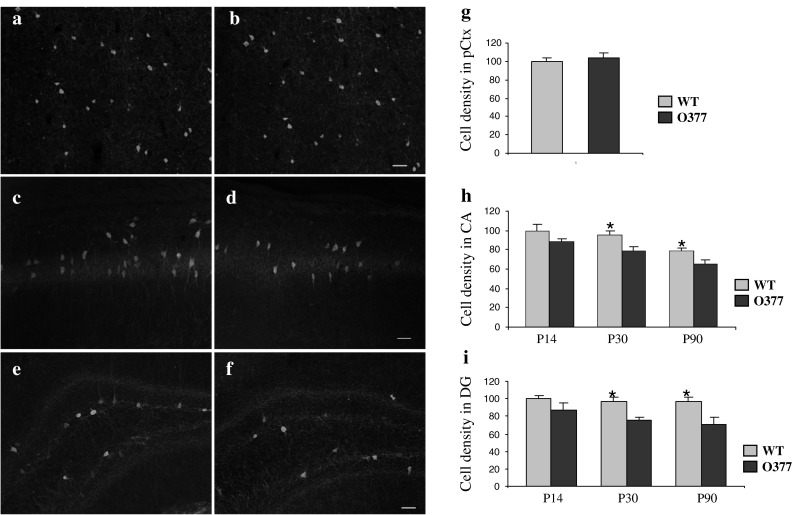
Reduced number of parvalbumin-positive interneurons in the hippocampus of *O377* mutant mice. The cell density of parvalbumin-positive interneurons in the DG and in the ventral CA is compared between wild-type mice and *O377* mutants at different ages. **a**, **b** Parvalbumin-positive GABAergic interneurons in the prefrontal cortex (pCtx) of 3-month-old wild type (**a**) and *O377* mutant mice (**b**). **c**, **d** Parvalbumin-positive GABAergic interneurons in the ventral CA1 of 3-month-old wild-type (**c**) and *O377* mutant mice (**d**). **e**, **f** Parvalbumin-positive GABAergic interneurons in the ventral DG of 3-month-old wild-type (**e**) and *O377* mutant mice (**f**). **g** Cell density of parvalbumin-positive GABAergic interneurons in the prefrontal cortex of 3-month-old wild-type and *O377* mutant mice. **h** Cell density of parvalbumin-positive GABAergic interneurons in the CA of wild-type and *O377* mutant mice between 2 weeks (P14) and 3 months (P90) of age. **i** Cell density of parvalbumin-positive GABAergic interneurons in the DG of wild-type and *O377* mutant mice between 2 weeks (P14) and 3 months (P90) of age. In **g**–**i**, values are normalized to wild-type values at the youngest age and given in % (WT = 100 %). *p < 0.05; Student’s *t* test, *n* ≥ 4. *Scale* *bar* 40 μm; *error bar* SEM

We next asked whether distinct interneuronal subpopulations might be altered in the hippocampus of *O377* mutants. Indeed, we found that the cell density of parvalbumin-positive neurons in the ventral CA and DG of 3-month old *O377* mutants was significantly lower than that in wild-type mice (Fig. 5). This is likely due to a slow process, with a decreasing trend already visible in the first postnatal weeks leading to a progressively increasing difference between WT mice and mutants (Fig. 5h, i). In contrast, the parvalbumin population was not affected in the prefrontal cortex (Fig. 5g). Likewise, the density of calretinin- and somatostatin-positive neurons in the prefrontal cortex, ventral CA, and ventral DG was not significantly different between 3-month-old WT and *O377* mutant mice (Supplementary Figs. 10, 11).

### Increased translation of input-to-output neuronal activity in the DG of *O377* mutants

**Fig. 6:**
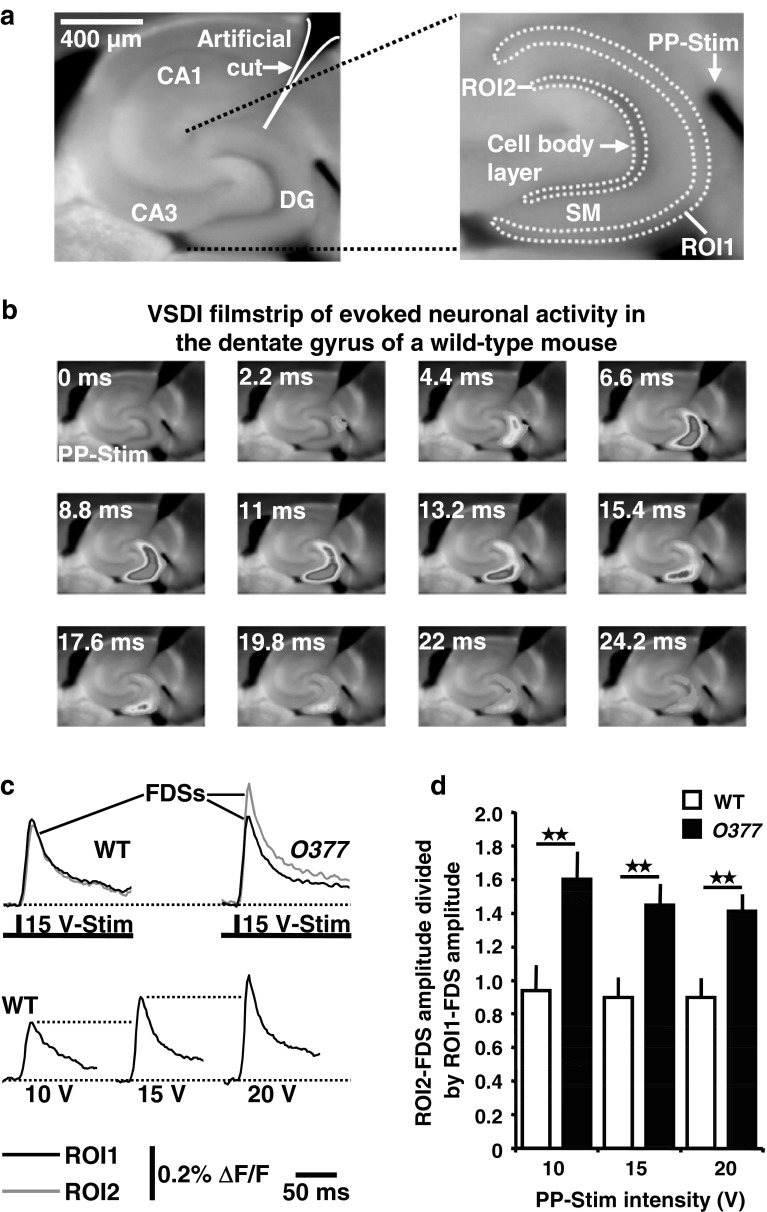
Altered functionality of the DG in *O377* mutants. **a** Experimental arrangement used for the VSDI experiments depicted and quantified in **b**–**d**. The artificial cut was made to prevent CA1 neuronal activity resulting from action potentials in fibers of the temporoammonic pathway. **b** VSDI filmstrip of the spread of neuronal activity evoked by a single electrical stimulation pulse (200 μs, 15 V) delivered via an extracellular electrode to the perforant path. Warmer colors represent stronger neuronal activity. **c** VSDI recording traces from two of the in **d** quantified experiments. As the VSDI measure of neuronal activity, we used ROI-extracted, fast, depolarization-mediated imaging signals (FDSs; see also the “Materials and methods” section). **d** Increased translation of input (ROI1-FDS)-to-output (ROI2-FDS) neuronal activity in the DG of *O377* mutants (for each condition, *n* = 8 slices from 4 mice). Results are presented as mean ± SEM. **p < 0.01 (unpaired *t* test)

We investigated whether the phenotypes discussed above correlate with altered hippocampal functionality. For this purpose, we performed electrophysiological measurements in ventral hippocampal brain slices from 13 to 17-week-old male mice. In the first set of experiments, we tested for differences in the frequency of GABA_A_ receptor-mediated mIPSCs in DG granule cells between *O377* mutants and wild-type mice; however, these experiments revealed no statistically significant differences in the frequency (and amplitude) of mIPSCs (Supplementary Fig. 12). Therefore, we considered that functional changes can be uncovered only at the level of neuronal activity of the whole network. To test this, we electrically triggered glutamatergic synaptic transmission at perforant path projections to DG neurons and conducted high-speed voltage-sensitive dye imaging (Airan et al. 2007; von Wolff et al. 2011) of the resultant neuronal activity. By means of a ROI operation, we quantified the neuronal depolarization (“activity”) strength in the region of perforant path synaptic input (outer third of *stratum moleculare*, ROI1) and the region where the cell bodies of granule cells are located (defined as output region, ROI2). This was done since we hypothesized that a decreased number of inhibitory interneurons should cause an increased translation of input-to-output neuronal activity in the DG of *O377* mutants. As the final measure, we divided the neuronal depolarization strength within ROI2 by the neuronal depolarization strength within ROI1. Consistent with our hypothesis, we found this quotient to be markedly enhanced in *O377* mutants. This mutation effect was independent of the strength of perforant path electrical stimulation (Fig. 6a–d).

## Discussion

Although βB2-crystallin (*Crybb2, CRYBB2*) was first described as a structural protein of the ocular lens, further roles for this gene are expected given its expression in the brain. In our basic study (Ganguly et al. 2008), we observed increased expression of the gene encoding calpain 3 (*Capn3*). Therefore, we used this finding, together with an initial behavioral analysis, as a starting point for functional analysis of the effect of the underlying *Crybb2* mutation on the brain.

The behavioral analysis revealed two significant results: increased activity of the mutants in social investigation and a decreased ASR in combination with an increased PPI (Fig. 1). Since the increased social investigation was not associated with an alteration in olfaction, we did not follow this line any further. However, the increased PPI in the *O377* mouse mutants is an intriguing coincidence with results of a previous report that mapped a PPI-QTL to mouse chromosome 5, roughly between 45 and 66 cM (Joober et al. 2002) where the *Crybb2* gene is mapped.

The hippocampus is one of the areas of the brain where abundant *Crybb2* expression has previously been observed (Ganguly et al. 2008). Moreover, by repeated inspection of brain sections, we realized that the hippocampus of the *O377* mutants seemed to be smaller. Detailed measurements confirmed the impression and motivated us to focus our studies on the hippocampus. The smaller size of the hippocampus might be explained at least in part by apoptosis in the hippocampus of young mutants. Differences in apoptosis are not observed in adults, and other possible mechanisms (e.g., decreased neurogenesis or impaired neuronal maturation) are not supported by our data (Fig. 2; Supplementary Figs. 5, 6). Increased apoptosis in the mutants might be due to the increased basal concentration of Ca^2+^ (Fig. 3).

Nevertheless, apoptosis does not explain the specific loss of parvalbumin-positive interneurons in the hippocampus of *O377* mutants (Fig. 5); for this feature and its physiological consequences (increased input:output ratio in the DG), we have elaborated on another series of events; however, it may also start from the enhanced level of free Ca^2+^ in the mutants. The mechanisms by which the expression of *Crybb2* translates into a neuronal subtype-specific phenotype might be explained better by the increased *Capn3* expression (Fig. 4h) based upon the calcium-binding domains of the mouse *Capn3* gene (Herasse et al. 1999). This increased *Capn3* expression might lead to the decreased number of NMDA receptors (Fig. 4i–l) and finally to the loss of parvalbumin-positive interneurons. Even if the data reported here can be organized in a putative series of events, we are not yet in a position to define each step involved in this process. It also includes the caveat that changes in the expression level might not lead immediately to changes at the protein level.

The first and central point in our model is the Ca^2+^-binding activity of βB2-crystallin, which had been demonstrated earlier (Jobby and Sharma 2007; Aravind et al. 2009). Unfortunately, we could not test the loss of Ca^2+^-binding activity of the mutated βB2-crystallin, because the corresponding recombinant protein remains insoluble in several buffer systems (M. Sun, unpublished observation). However, the observed increase of free Ca^2+^ (Fig. 3) is in agreement with the reported Ca^2+^-binding properties of βB2-crystallin (Jobby and Sharma 2007). In an evolutionary context, Ca^2+^ binding by βB2-crystallin might be its original function, and, like other crystallins, βB2-crystallin might have been recruited to the lens to modify its properties (for a recent review see Wistow 2012).

Since *Crybb2* is expressed during embryonic development in the brain (from E14.5 onward; Supplementary Fig. 2), it is very likely that the first alterations can appear early in life. Therefore, the increased concentration of free Ca^2+^, which has been observed in extracts from embryonic hippocampi of mutants, may have at least two consequences: increased apoptosis and increased *Capn3* expression. Since the increased apoptosis in the hippocampus of the mutant mice during the first 3 months after birth is evident (Fig. 2), the means by which increased Ca^2+^ levels may lead to an increased expression of *Capn3* [as observed previously by Ganguly et al. (2008) in a total-brain mRNA pool and particularly in the hippocampus as demonstrated here in Fig. 3] is not immediately obvious. This link might be mediated by a protein being able to measure the actual concentration of free Ca^2+^. Such a protein is well known as Ca^2+^-sensing receptor (CaSR), a G protein–coupled receptor. In situ hybridization, histochemistry, and immunohistochemistry revealed CaSR mRNA and protein in pyramidal cells of all the layers of the hippocampus and in granule cells of the DG of the rat (Chattopadhyay et al. 1997). The highest levels are present within the subfornical organ and in the olfactory bulb. Substantial levels of expression are also evident within the striatum, cingulate cortex, cerebellum, ependymal zones of the cerebral ventricles, and perivascular nerves around cerebral arteries (Yano et al. 2004), but there are no reports dealing with its expression in the cortex. Finally, the involvement of CaSR in the transcription of neuronal genes has also been discussed (Riccardi and Kemp 2012).

The enhanced *Capn3* gene expression, as reported previously using expression arrays (Ganguly et al. 2008) and validated here (Fig. 4h), may lead to a downregulation of NMDA receptor subtype gene expression (for review see Wu and Lynch 2006), as observed here 2 weeks after birth (Fig. 4i–l; P14). Additionally, it is well established that increased calpain activity leads to a degradation of NMDA receptors (Bi et al. 1998; Araújo et al. 2005). In turn, hypofunction of NMDA receptors has been associated with a loss of GABAergic interneurons, especially parvalbumin-positive GABAergic interneurons (Lodge et al. 2009; Romón et al. 2011; Gandal et al. 2012; Gonzalez-Burgos and Lewis 2012). This particular feature—upregulation of *Capn3* and downregulation of NMDA receptor gene expression—might explain the specific loss of parvalbumin-positive interneurons observed in the *O377* mutants.

The functional cornerstone in our experiments reported here is the increased translation of input-to-output neuronal activity in the DG of the *O377* mutants (Fig. 6). It indicates that the morphological changes observed have functional consequences for the neuronal network of the hippocampus and might explain the increase in PPI. PPI is regulated largely by neuronal connections between the limbic cortex (including the entorhinal cortex, hippocampus, and amygdala), ventral striatum, ventral pallidum, and pontine tegmentum. Glutamatergic fibers from the limbic cortex converge at the nucleus accumbens within the PPI regulatory circuitry. GABAergic projections, originating in the nucleus accumbens, then project to the globus pallidus. Regulation of PPI is carried out by GABAergic projections from the globus pallidus to the pedunculopontine nucleus. Increased PPI is associated with a reduction of GABAergic projections from the globus pallidus, while decreases are associated with the reverse (see Geyer et al. 2001 for review; Swerdlow et al. 2001). Thus, the increased translation of input-to-output neuronal activity in the DG of *O377* mutants, as determined by the electrophysiological measurements, could lead to enhanced accumbal GABAergic activation, resulting in increased inhibition of the GABAergic neurons of the globus pallidus, thereby disinhibiting the pedunculopontine nucleus, which ultimately increases PPI.

While there is substantial evidence for a functional impairment of GABAergic inhibitory interneurons in the hippocampus in schizophrenia, as reviewed in Powell et al. (2009), there is hardly any evidence in the literature directly linking the loss of parvalbumin-positive neurons in the hippocampus to an increase in PPI. It has been suggested that NMDA receptor hypofunction and alterations in the parvalbumin-positive interneurons are involved in schizophrenia in mice (Lodge et al. 2009) and in humans (Gonzalez-Burgos and Lewis 2012), but PPI was not measured in these studies. Forebrain-specific glutamate receptor B deletion in the mouse resulted in a decrease of parvalbumin-expressing interneurons in the DG and the pyramidal cells in CA3 and decreased neurogenesis in the subgranular zone, altered excitatory synaptic transmission in the hippocampus, and impaired spatial memory, but PPI was also not assessed in this study (Shimshek et al. 2006). In rats, isolation housing induced a reduction in PPI and action potential height in subicular pyramidal neurons as well as an increase in calretinin-positive neurons in DG, but hippocampal parvalbumin immunoreactivity remained unchanged (Greene et al. 2001). However, selective lesioning of septohippocampal GABAergic neurons in rats was associated with reduced parvalbumin immunoreactivity in the septal area and an increase in PPI (Ma et al. 2012).

While PPI deficits are believed to have face, construct, and a high predictive validity for schizophrenia in man (Swerdlow et al. 1994; Geyer et al. 2001), the biological relevance of increased PPI is less clear. One study suggested that in healthy humans, elevated PPI reflects enhanced preattentive perceptual processing and is associated with improved performance in upstream cognitive functions [measured by 5-choice reaction time tests, problem-solving tasks, and a test of spatial working memory (Bitsios et al. 2006)]. This was exemplified by enhanced strategy formation and execution times, possibly due to more efficient early information processing. As problem solving is determined by the integrity of the frontal lobe, increased PPI may signify altered prefrontal lobe function. In contrast, enhancement of PPI was also observed at a prodromal stage in a tauopathy mouse model of Alzheimer’s disease, and the authors suggested using the assessment of sensorimotor gating to detect the earliest manifestations of tauopathies (Takeuchi et al. 2011). In clinically healthy humans, no clear adaptive or functional advantage of higher versus lower levels of PPI is known, and it was suggested to be simply used as a “surrogate measure of neural processes” (Swerdlow et al. 2008).

## Conclusions

Our study demonstrated for the first time an association between sensorimotor gating and hippocampal function with a mutation in *Crybb2*. These potentially important findings need further validation and additional work from different disciplines to clarify the mechanisms involved. Nevertheless, these results suggest an interesting novel avenue for further exploration and offer a first functional model for the action of βB2-crystallin in the brain.

## Electronic supplementary material

Below is the link to the electronic supplementary material.

Supplementary material 1 (PPT 9607 kb)

## References

[CR1] Airan RD, Meltzer LA, Roy M, Gong Y, Chen H, Deisseroth K (2007). High-speed imaging reveals neurophysiological links to behavior in an animal model of depression. Science.

[CR2] Araújo IM, Xapelli S, Gil JM, Mohapel P, Petersén A, Pinheiro PS, Malva JO, Bahr BA, Brundin P, Carvalho CM (2005). Proteolysis of NR2B by calpain in the hippocampus of epileptic rats. Neuroreport.

[CR3] Aravind P, Mishra A, Suman SK, Jobby MK, Sankaranarayanan R, Sharma Y (2009). The bg-crystallin superfamily contains a universal motif for binding calcium. Biochemistry.

[CR4] Bi X, Rong Y, Chen J, Dang S, Wang Z, Baudry M (1998). Calpain-mediated regulation of NMDA receptor structure and function. Brain Res.

[CR5] Bitsios P, Giakoumaki SG, Theou K, Frangou S (2006). Increased prepulse inhibition of the acoustic startle response is associated with better strategy formation and execution times in healthy males. Neuropsychologia.

[CR6] Boldrini M, Underwood MD, Hen R, Rosoklija GB, Dwork AJ, John Mann J, Arango V (2009). Antidepressants increase neural progenitor cells in the human hippocampus. Neuropsychopharmacology.

[CR7] Chattopadhyay N, Legradi G, Bai M, Kifor O, Ye C, Vassilev PM, Brown EM, Lechan RM (1997). Calcium-sensing receptor in the rat hippocampus: a developmental study. Brain Res Dev Brain Res.

[CR8] Díaz-Hernández M, Pereira MF, Pintor J, Cunha RA, Ribeiro JA, Miras-Portugal MT (2002). Modulation of the rat hippocampal dinucleotide receptor by adenosine receptor activation. J Pharmacol Exp Ther.

[CR9] Duprey KM, Robinson KM, Wang Y, Taube JR, Duncan MK (2007). Subfertility in mice harboring a mutation in βB2-crystallin. Mol Vis.

[CR10] Ehm O, Göritz C, Covic M, Schäffner I, Schwarz TJ, Karaca E, Kempkes B, Kremmer E, Pfrieger FW, Espinosa L, Bigas A, Giachino C, Taylor V, Frisén J, Lie DC (2010). RBPJκ-dependent signaling is essential for long-term maintenance of neural stem cells in the adult hippocampus. J Neurosci.

[CR11] Feil R, Hölter SM, Weindl K, Wurst W, Langmesser S, Gerling A, Feil S, Albrecht U (2009). cGMP-dependent protein kinase I, the circadian clock, sleep, and learning. Commun Integr Biol.

[CR12] Gandal MJ, Sisti J, Klook K, Ortinski PI, Leitman V, Liang Y, Thieu T, Anderson R, Pierce RC, Jonak G, Gur RE, Carlson G, Siegel SJ (2012). GABAB-mediated rescue of altered excitatory-inhibitory balance, gamma synchrony and behavioral deficits following constitutive NMDAR-hypofunction. Transl Psychiatry.

[CR13] Ganguly K, Favor J, Neuhäuser-Klaus A, Sandulache R, Puk O, Beckers J, Horsch M, Schädler S, Vogt Weisenhorn D, Wurst W, Graw J (2008). Novel allele of *Crybb2* in the mouse and its expression in the brain. Invest Ophthalmol Vis Sci.

[CR14] Geyer MA, Krebs-Thomson K, Braff DL, Swerdlow NR (2001). Pharmacological studies of prepulse inhibition models of sensorimotor gating deficits in schizophrenia: a decade in review. Psychopharmacology.

[CR15] Gonzalez-Burgos G, Lewis DA (2012). NMDA receptor hypofunction, parvalbumin-positive neurons and cortical gamma oscillations in schizophrenia. Schizophr Bull.

[CR16] Graw J (2009). Crystallins: cataract and beyond. Exp Eye Res.

[CR17] Greene JRT, Kerkhoff JE, Guiver L, Totterdell S (2001). Structural and functional abnormalities of the hippocampal formation in rats with environmentally induced reductions in prepulse inhibition of acoustic startle. Neuroscience.

[CR18] Grynkiewicz G, Poenie M, Tsien RY (1985). A new generation of Ca^2+^ indicators with greatly improved fluorescence properties. J Biol Chem.

[CR19] Haupt C, Witte OW, Frahm C (2007). Temporal profile of connexin 43 expression after photothrombotic lesion in rat brain. Neuroscience.

[CR20] Herasse M, Ono Y, Fougerousse F, Kimura E, Stockholm D, Beley C, Montarras D, Pinset C, Sorimachi H, Suzuki K, Beckmann JS, Richard I (1999). Expression and functional characteristics of calpain 3 isoforms generated through tissue-specific transcriptional and posttranscriptional events. Mol Cell Biol.

[CR21] Huang CH, Wang YT, Tsai CF, Chen YJ, Lee JS, Chiou SH (2011). Phosphoproteomics characterization of novel phosphorylated sites of lens proteins from normal and cataractous human eye lenses. Mol Vis.

[CR22] Jobby MK, Sharma Y (2007). Calcium-binding to lens betaB2- and betaA3-crystallins suggests that all beta-crystallins are calcium-binding proteins. FEBS J.

[CR23] Joober R, Zarate JM, Rouleau GA, Skamene E, Boksa P (2002). Provisional mapping of quantitative trait loci modulating the acoustic startle response and prepulse inhibition of acoustic startle. Neuropsychopharmacology.

[CR24] Kleiman NJ, Chiesa R, Kolks MA, Spector A (1988). Phosphorylation of β-crystallin B2 (βBp) in the bovine lens. J Biol Chem.

[CR25] Kubota Y, Shigematsu N, Karube F, Sekigawa A, Kato S, Yamaguchi N, Hirai Y, Morishima M, Kawaguchi Y (2011). Selective coexpression of multiple chemical markers defines discrete populations of neocortical GABAergic neurons. Cereb Cortex.

[CR26] Liedtke T, Schwamborn JC, Schroer U, Thanos S (2007). Elongation of axons during regeneration involves retinal crystallin beta b2 (Crybb2). Mol Cell Proteomics.

[CR27] Lodge DJ, Behrens MM, Grace AA (2009). A loss of parvalbumin-containing interneurons is associated with diminished oscillatory activity in an animal model of schizophrenia. J Neurosci.

[CR28] Ma J, Tai SK, Leung LS (2012). Septohippocampal GABAergic neurons mediate the altered behaviors induced by N-methyl-D-aspartate receptor antagonists. Hippocampus.

[CR29] Mandillo S, Tucci V, Hölter SM, Meziane H, Banchaabouchi MA, Kallnik M, Lad HV, Nolan PM, Ouagazzal AM, Coghill EL, Gale K, Golini E, Jacquot S, Krezel W, Parker A, Riet F, Schneider I, Marazziti D, Auwerx J, Brown SD, Chambon P, Rosenthal N, Tocchini-Valentini G, Wurst W (2008). Reliability, robustness and reproducibility in mouse behavioral phenotyping: a cross-laboratory study. Physiol Genomics.

[CR30] Ninkovic J, Pinto L, Petricca S, Lepier A, Sun J, Rieger MA, Schroeder T, Cvekl A, Favor J, Götz M (2010). The transcription factor Pax6 regulates survival of dopaminergic olfactory bulb neurons via crystallin αA. Neuron.

[CR31] Powell SB, Zhou X, Geyer MA (2009). Prepulse inhibition and genetic mouse models of schizophrenia. Behav Brain Res.

[CR32] Raynaud F, Marcilhac A (2006). Implication of calpain in neuronal apoptosis. A possible regulation of Alzheimer’s disease. FEBS J.

[CR33] Riccardi D, Kemp PJ (2012). The calcium-sensing receptor beyond extracellular calcium homeostasis: conception, development, adult physiology, and disease. Annu Rev Physiol.

[CR34] Romón T, Mengod G, Adell A (2011). Expression of parvalbumin and glutamic acid decarboxylase-67 after acute administration of MK-801. Implications for the NMDA hypofunction model of schizophrenia. Psychopharmacology.

[CR35] Shimshek DR, Jensen V, Celikel T, Geng Y, Schupp B, Bus T, Mack V, Marx V, Hvalby O, Seeburg PH, Sprengel R (2006). Forebrain-specific glutamate receptor B deletion impairs spatial memory but not hippocampal field long-term potentiation. J Neurosci.

[CR36] Swerdlow NR, Braff DL, Taaid N, Geyer MA (1994). Assessing the validity of an animal model of deficient sensorimotor gating in schizophrenic patients. Arch Gen Psychiatry.

[CR37] Swerdlow NR, Geyer MA, Braff DL (2001). Neural circuit regulation of prepulse inhibition of startle in the rat: current knowledge and future challenges. Psychopharmacology.

[CR38] Swerdlow NR, Weber M, Qu Y, Light GA, Braff DL (2008). Realistic expectations of prepulse inhibition in translational models for schizophrenia research. Psychopharmacology.

[CR39] Takeuchi H, Iba M, Inoue H, Higuchi M, Takao K, Tsukita K, Karatsu Y, Iwamoto Y, Miyakawa T, Suhara T, Trojanowski JQ, Lee VMY, Takahashi R (2011). P301S mutant human tau transgenic mice manifest early symptoms of human tauopathies with dementia and altered sensorimotor gating. PLoS One.

[CR40] Trinkl S, Glockshuber R, Jaenicke R (1994). Dimerization of βB2-crystallin: the role of the linker peptide and the N- and C-terminal extensions. Protein Sci.

[CR41] von Wolff G, Avrabos C, Stepan J, Wurst W, Deussing JM, Holsboer F, Eder M (2011). Voltage-sensitive dye imaging demonstrates an enhancing effect of corticotropin-releasing hormone on neuronal activity propagation through the hippocampal formation. J Psychiatr Res.

[CR42] Wall PM, Blanchard RJ, Yang M, Blanchard DC (2003). Infralimbic D2 receptor influences on anxiety-like behavior and active memory/attention in CD-1 mice. Prog Neuropsychopharmacol Biol Psychiatry.

[CR43] Wistow G (2012). The human crystallin gene families. Human Genomics.

[CR44] Wu HY, Lynch DR (2006). Calpain and synaptic function. Mol Neurobiol.

[CR45] Yano S, Brown EM, Chattopadhyay N (2004). Calcium-sensing receptor in the brain. Cell Calcium.

