# Supplementary material for: Crybb2 coding for βB2-crystallin affects sensorimotor gating and hippocampal function
Source: Mamm Genome. 2013 Oct 6;24(9):333–48. doi: 10.1007/s00335-013-9478-7 (PMC3824278; doi:10.1007/s00335-013-9478-7)
Supplement: Supplementary file 1 — Supplementary material 1 (PPT 9607 kb) [file 335_2013_9478_MOESM1_ESM.ppt]

## Slide 1
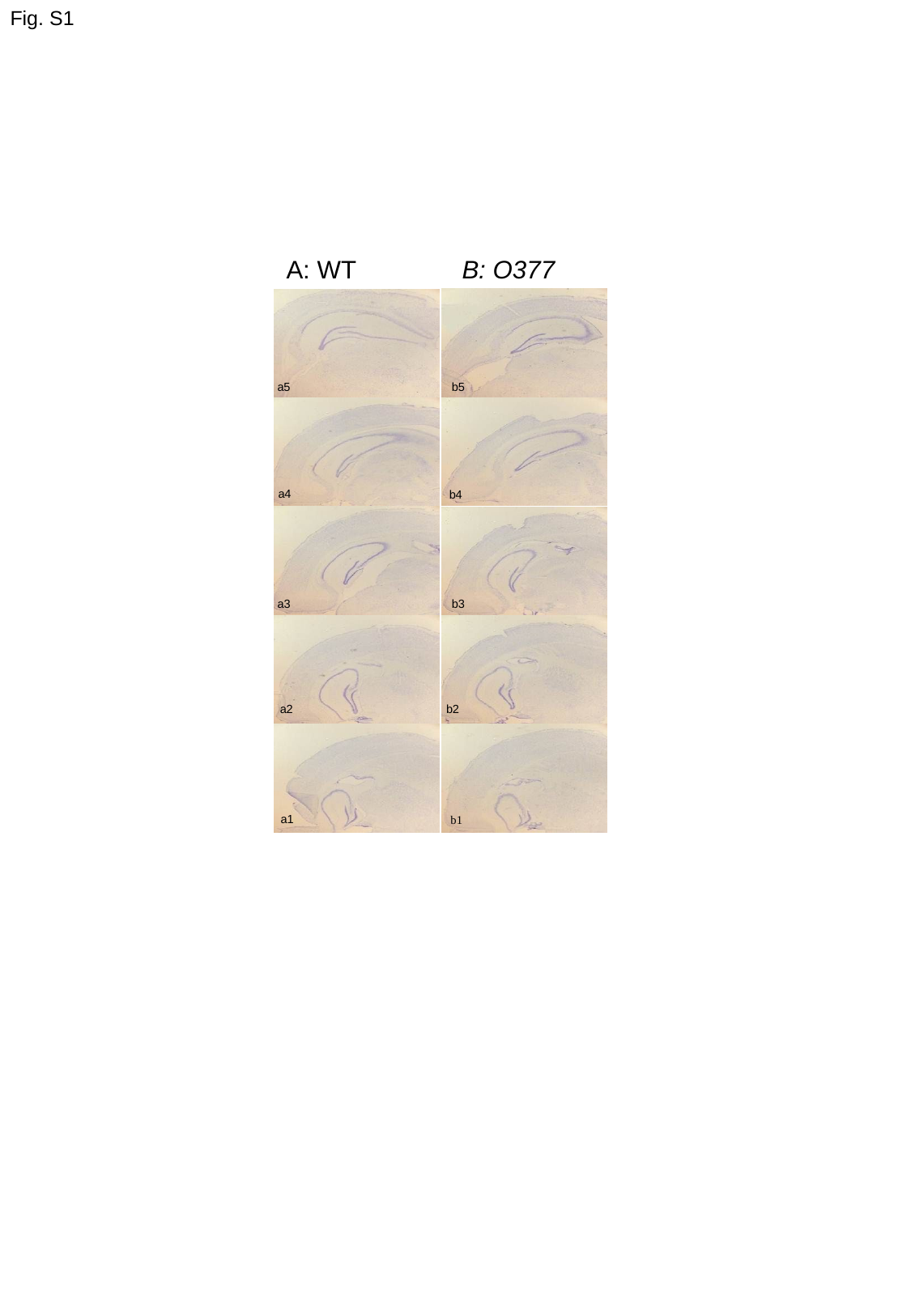

Fig. S1
A: WT B: O377
b5
a5
a4
b4
b3
a3
a2
b2
a1
b1

## Slide 2
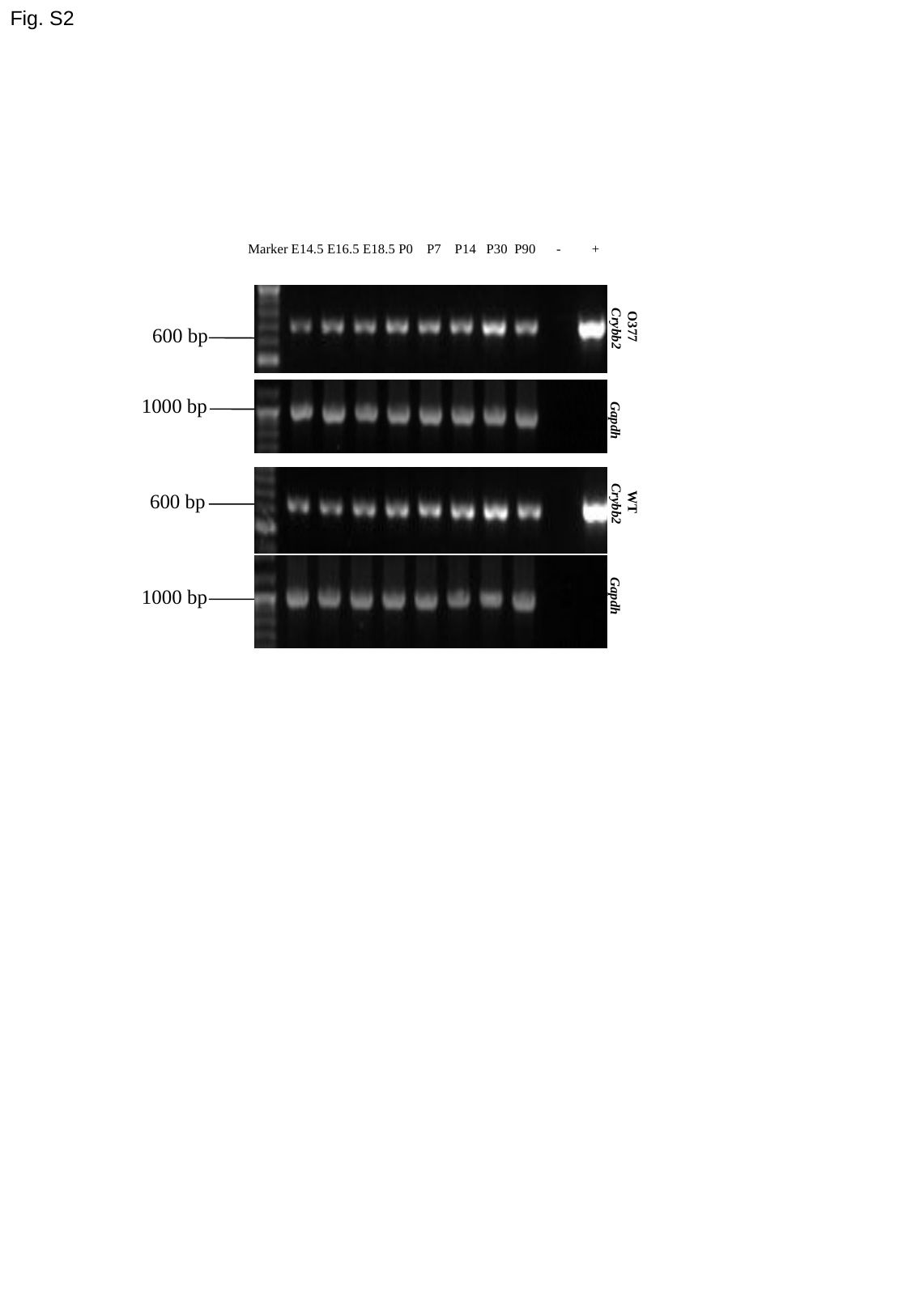

Fig. S2
 Marker E14.5 E16.5 E18.5 P0 P7 P14 P30 P90 - +
O377
Crybb2
C
600 bp
1000 bp
 Gapdh
600 bp
WT
Crybb2
1000 bp
 Gapdh
D
C

## Slide 3
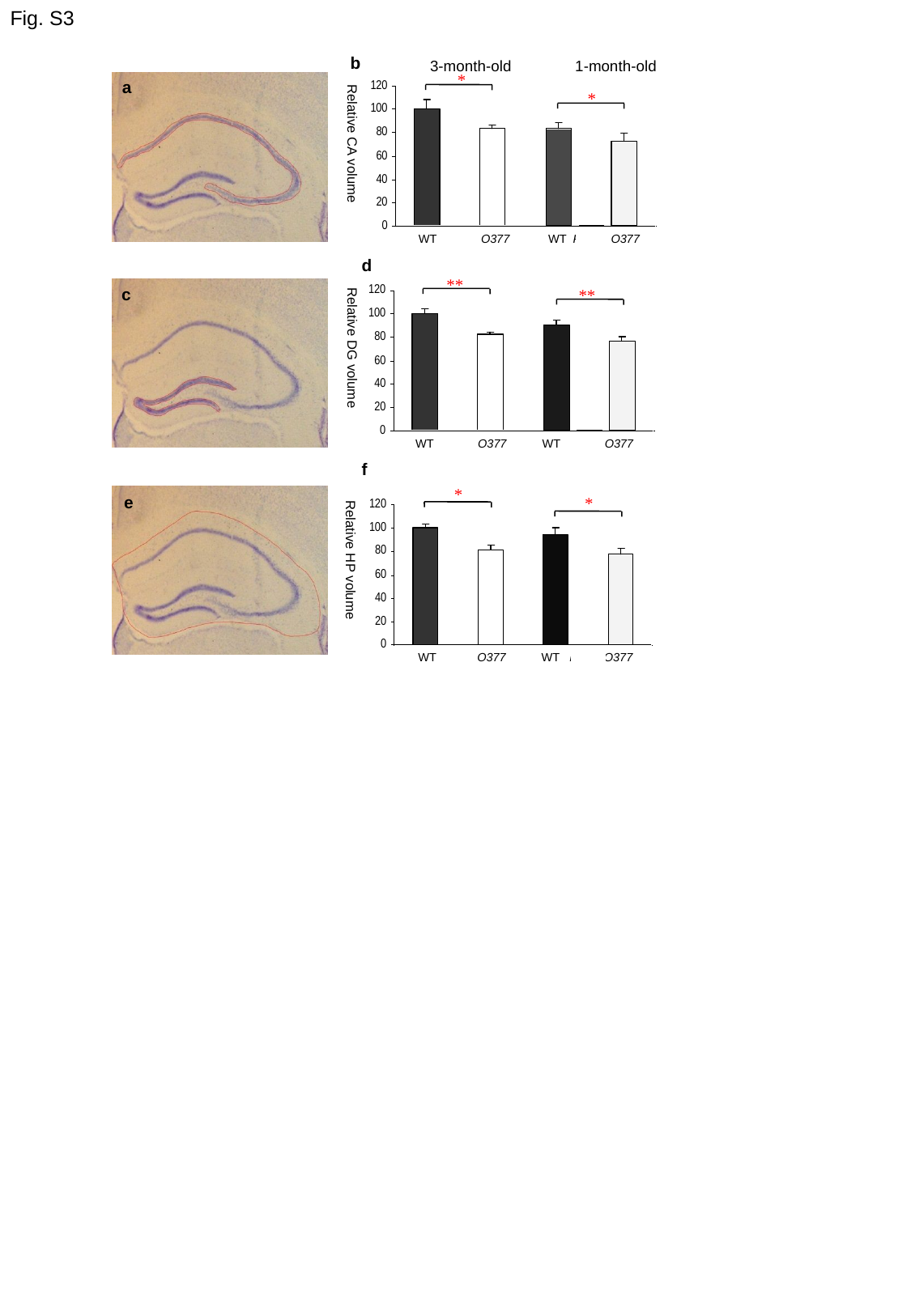

Fig. S3
b
3-month-old 1-month-old
 *
 *
a
Relative CA volume
 WT Philly O377 WT Philly O377
d
**
c
**
Relative DG volume
 WT Philly O377 WT Philly O377
f
 *
e
 *
Relative HP volume
 WT Philly O377 WT Philly O377

## Slide 4
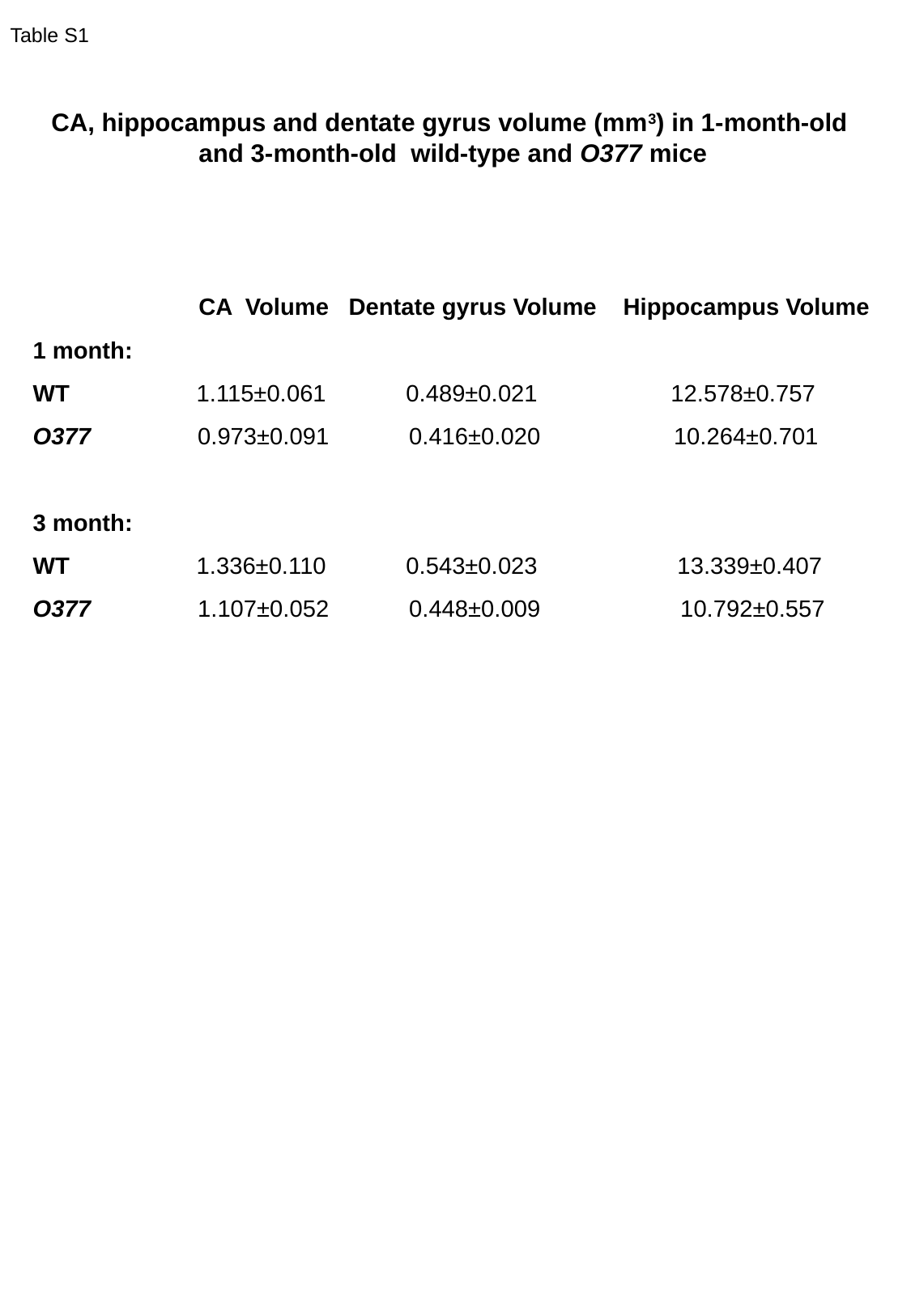

Table S1
CA, hippocampus and dentate gyrus volume (mm3) in 1-month-old and 3-month-old wild-type and O377 mice
	 CA Volume Dentate gyrus Volume Hippocampus Volume
1 month:
WT 1.115±0.061 0.489±0.021 12.578±0.757
O377 0.973±0.091 0.416±0.020 10.264±0.701
3 month:
WT 1.336±0.110 0.543±0.023 13.339±0.407
O377 1.107±0.052 0.448±0.009 10.792±0.557

## Slide 5
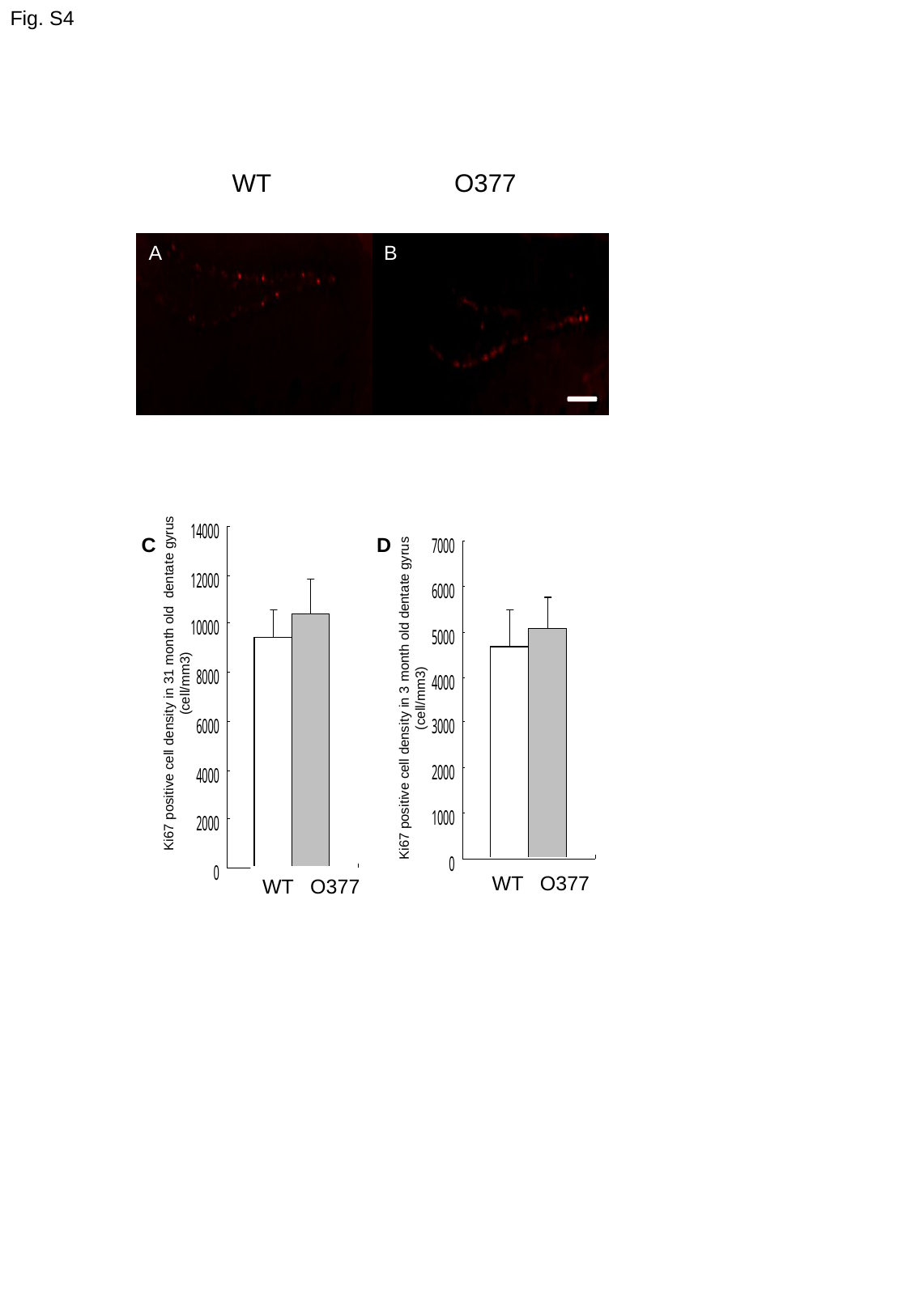

Fig. S4
WT O377
A
B
C
D
Ki67 positive cell density in 31 month old dentate gyrus (cell/mm3)
Ki67 positive cell density in 3 month old dentate gyrus (cell/mm3)
WT O377
WT O377

## Slide 6
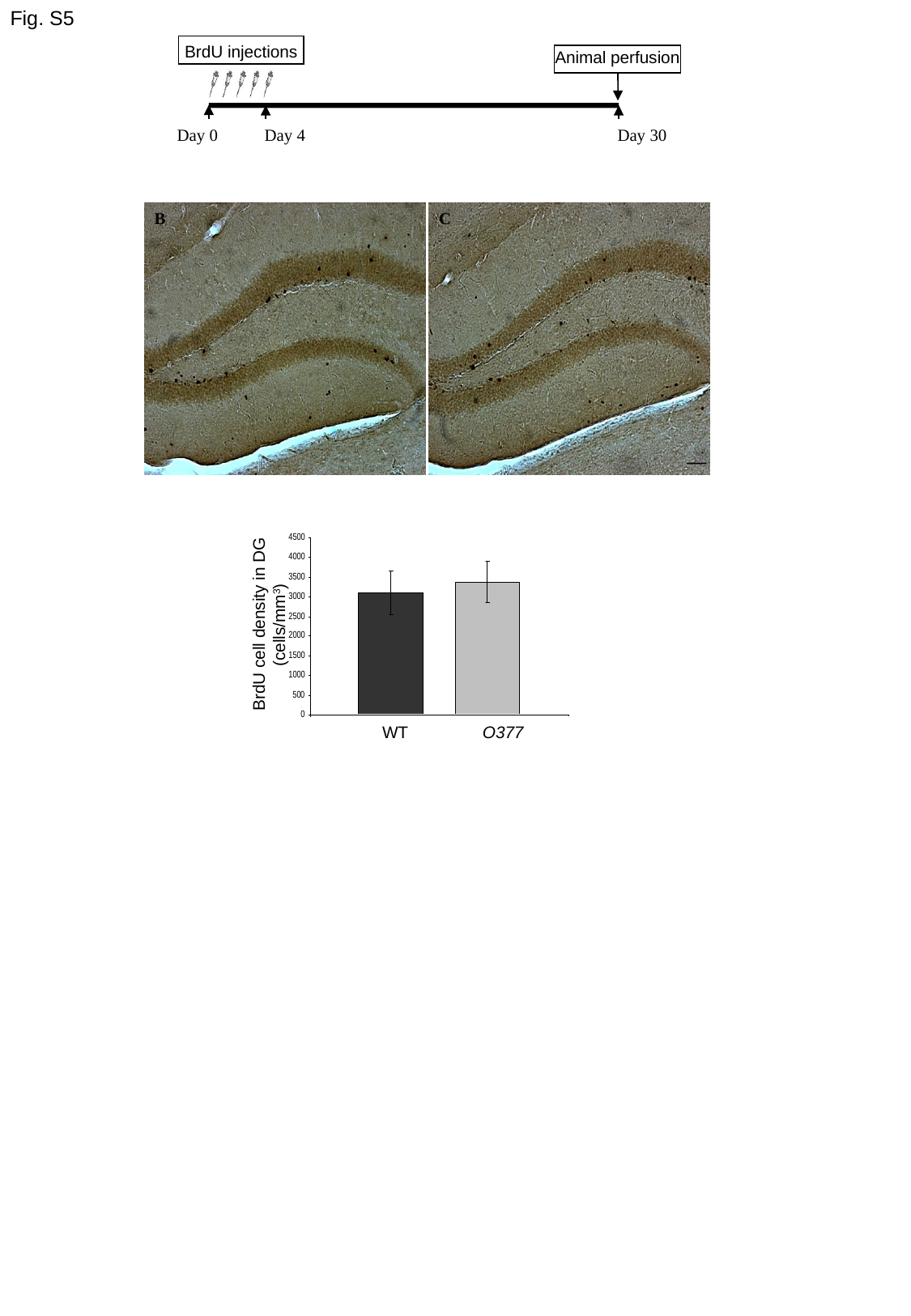

Fig. S5
BrdU injections
Animal perfusion
Day 0
Day 4
Day 30
B
C
 BrdU cell density in DG
(cells/mm3)
 WT O377

## Slide 7
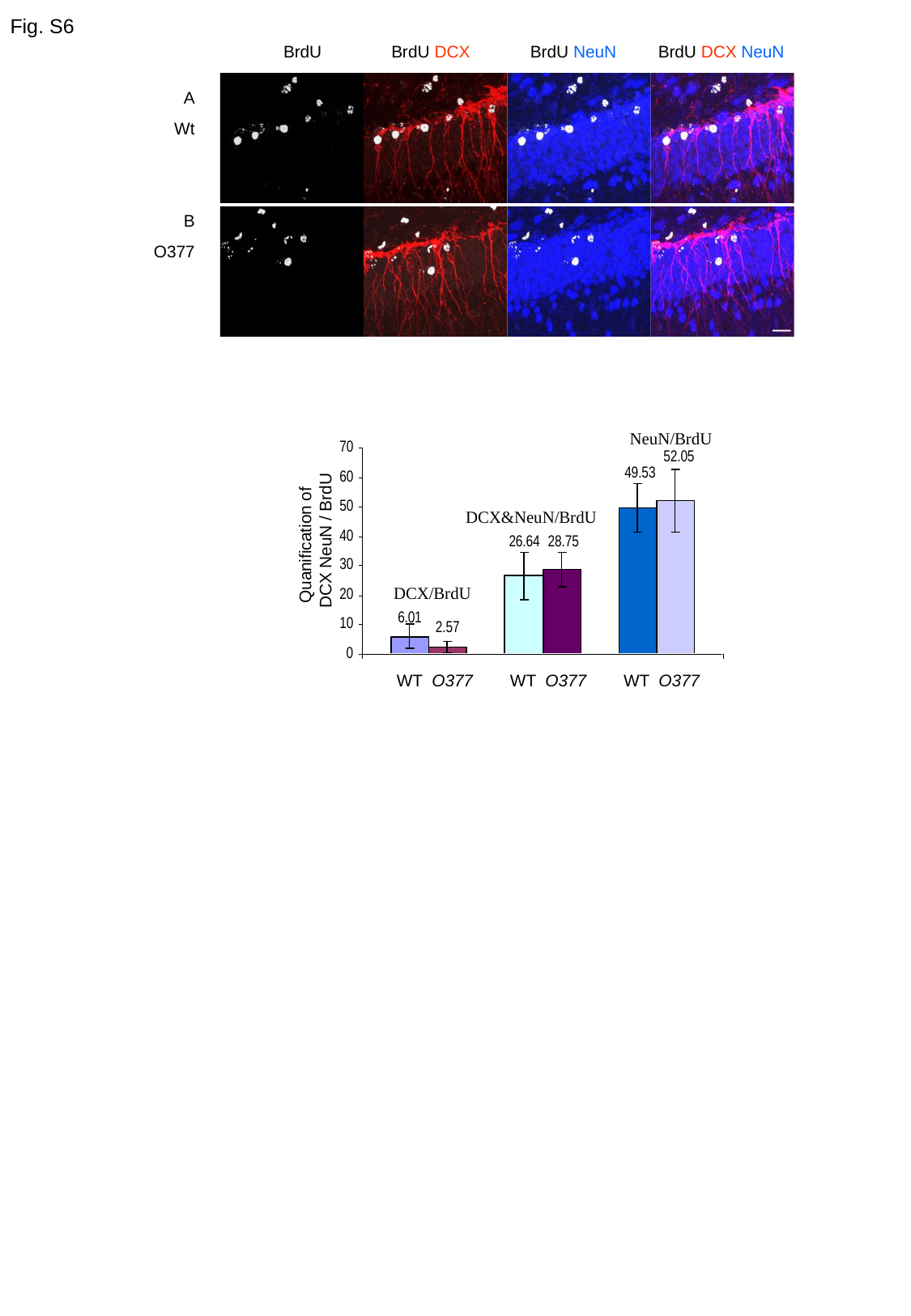

Fig. S6
BrdU BrdU DCX BrdU NeuN BrdU DCX NeuN
A
Wt
B
O377
 WT O377 WT O377 WT O377
NeuN/BrdU
DCX&NeuN/BrdU
Quanification of
 DCX NeuN / BrdU
DCX/BrdU

## Slide 8
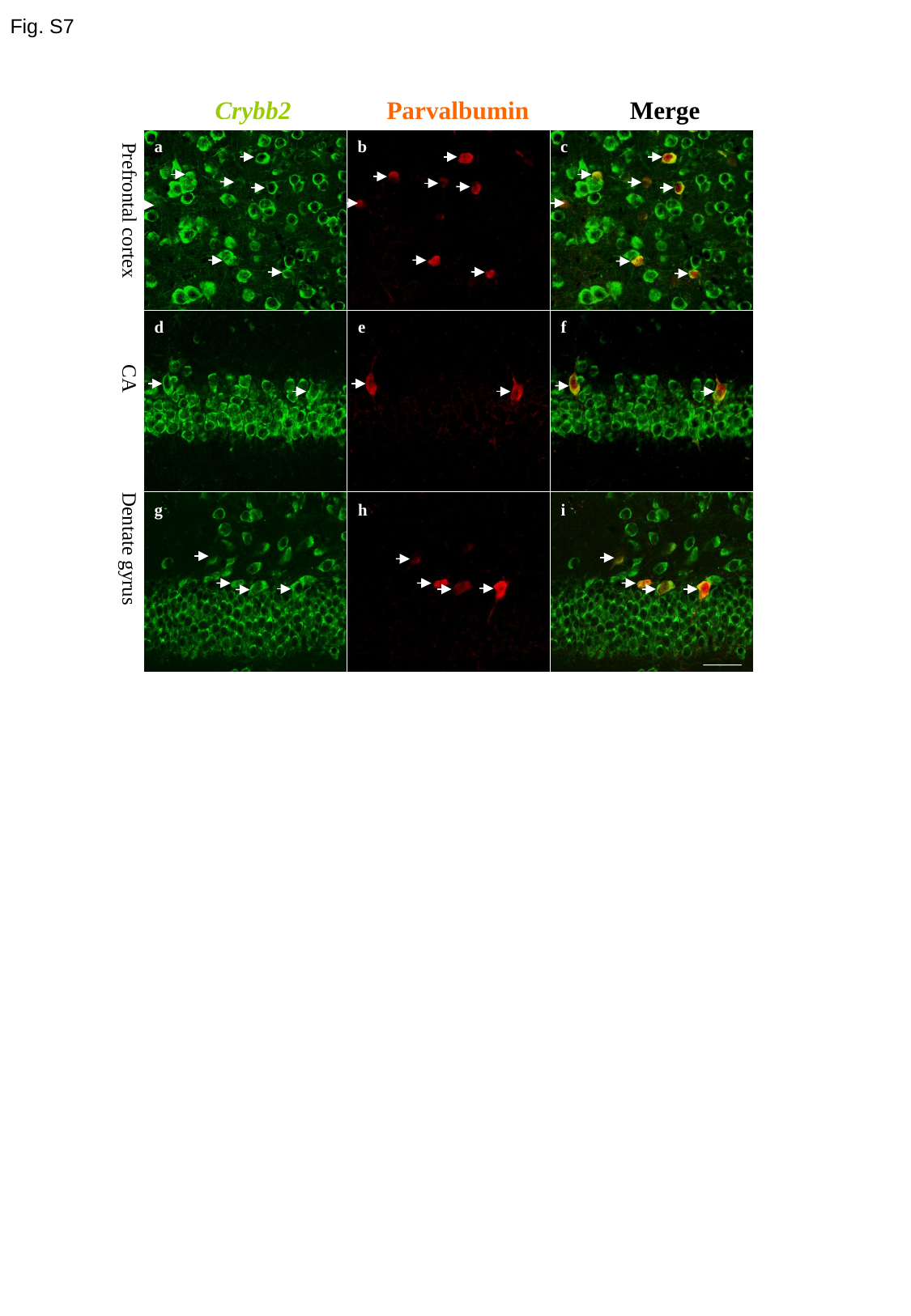

Fig. S7
 Crybb2 Parvalbumin Merge
 Prefrontal cortex CA Dentate gyrus
a
b
c
d
e
f
g
h
i

## Slide 9
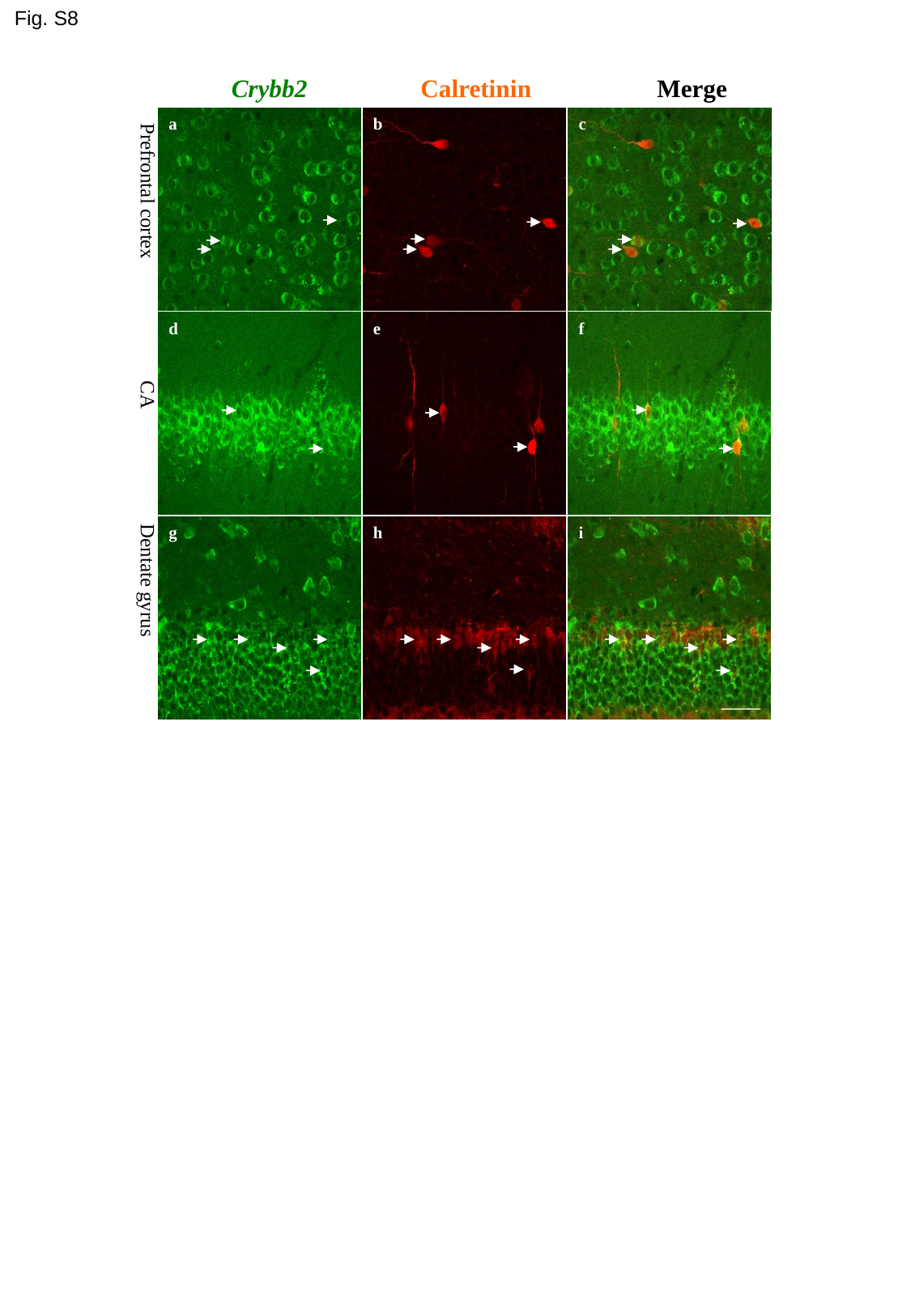

Fig. S8
 Crybb2 Calretinin Merge
 Prefrontal cortex CA Dentate gyrus
a
b
c
d
e
f
g
h
i

## Slide 10
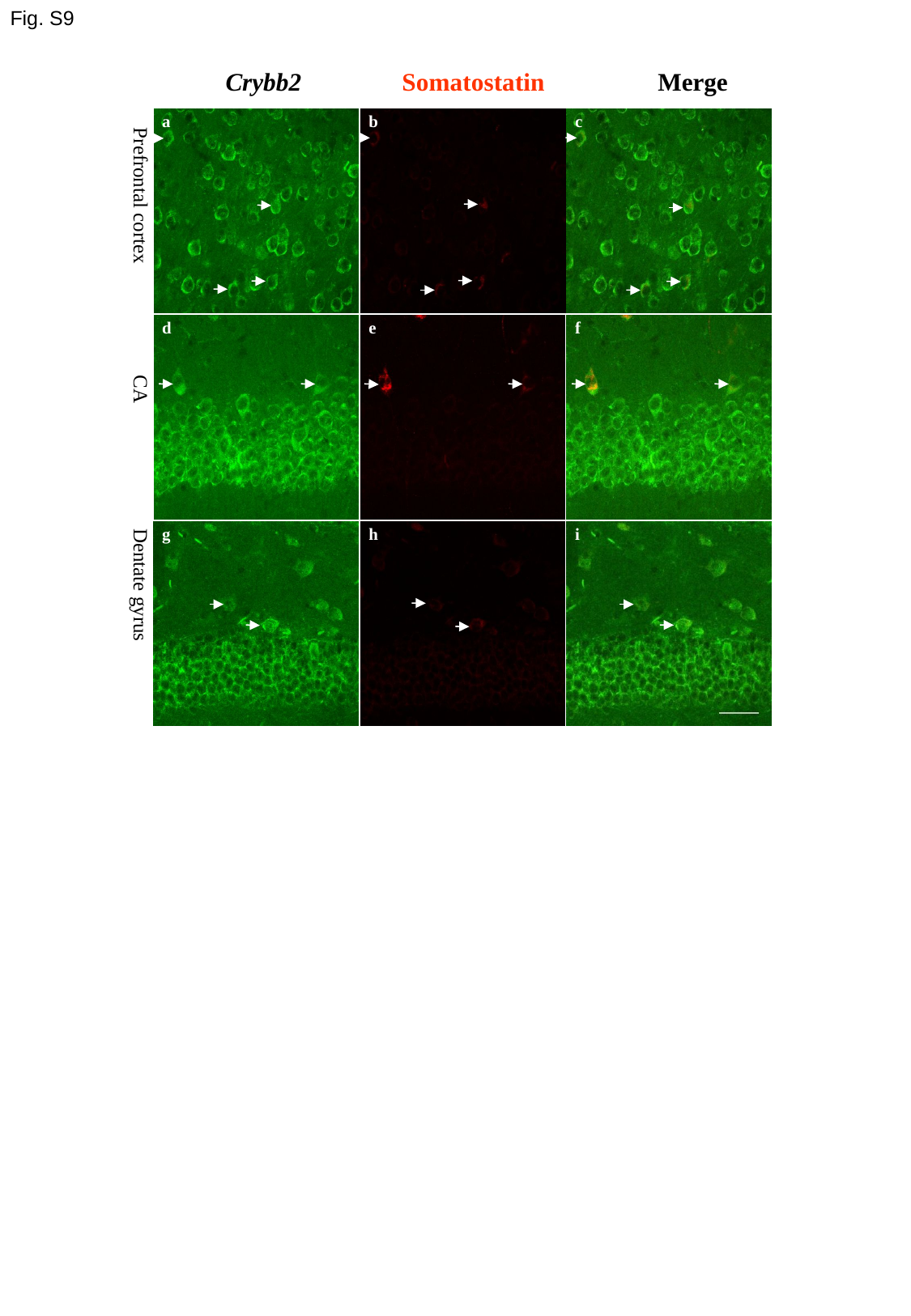

Fig. S9
 Crybb2 Somatostatin Merge
a
b
c
 Prefrontal cortex CA Dentate gyrus
d
e
f
g
h
i

## Slide 11
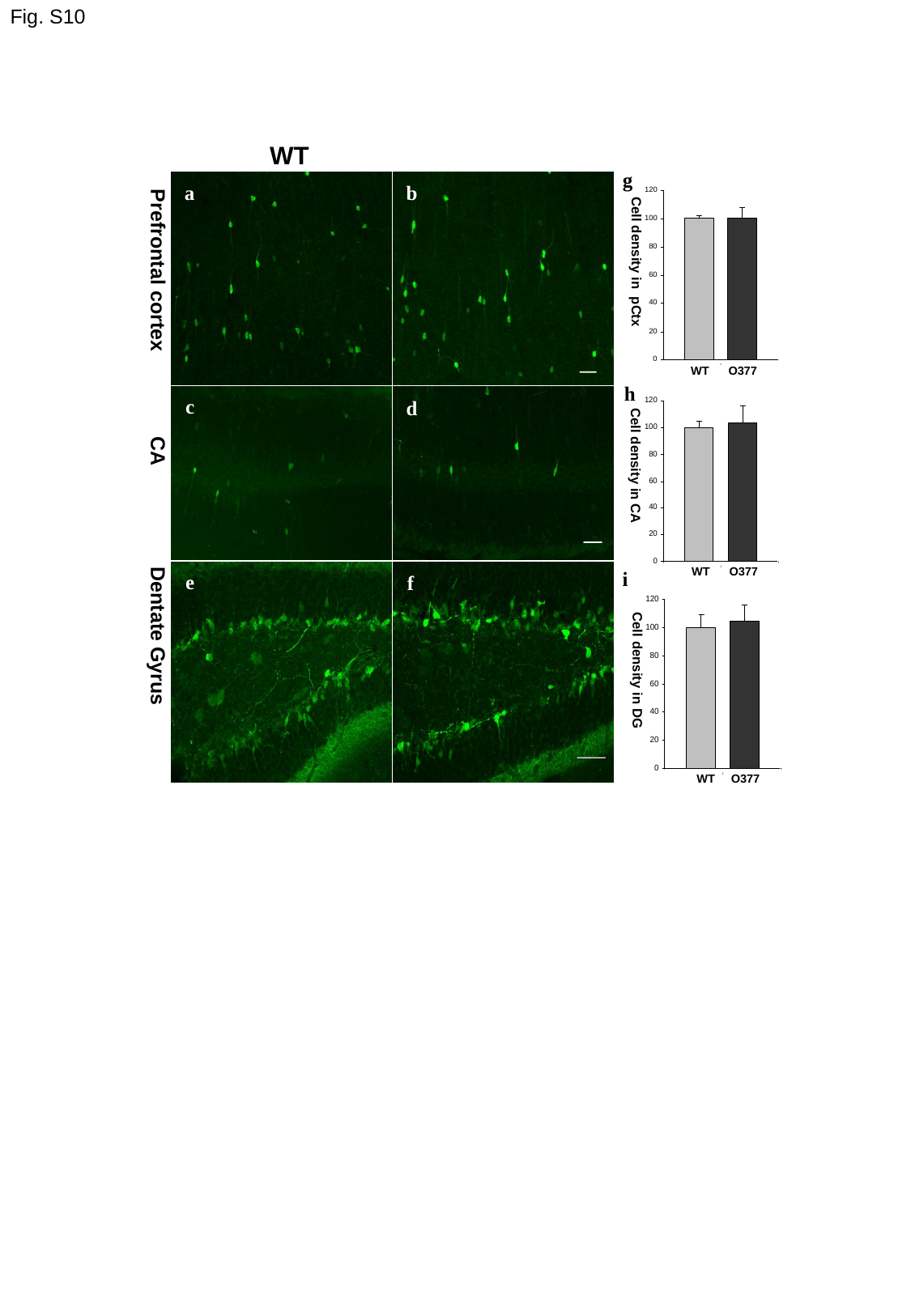

Fig. S10
WT O377
a
b
Prefrontal cortex CA Dentate Gyrus
c
d
e
f
g
Cell density in pCtx
WT O377
h
Cell density in CA
WT O377
i
Cell density in DG
WT O377

## Slide 12
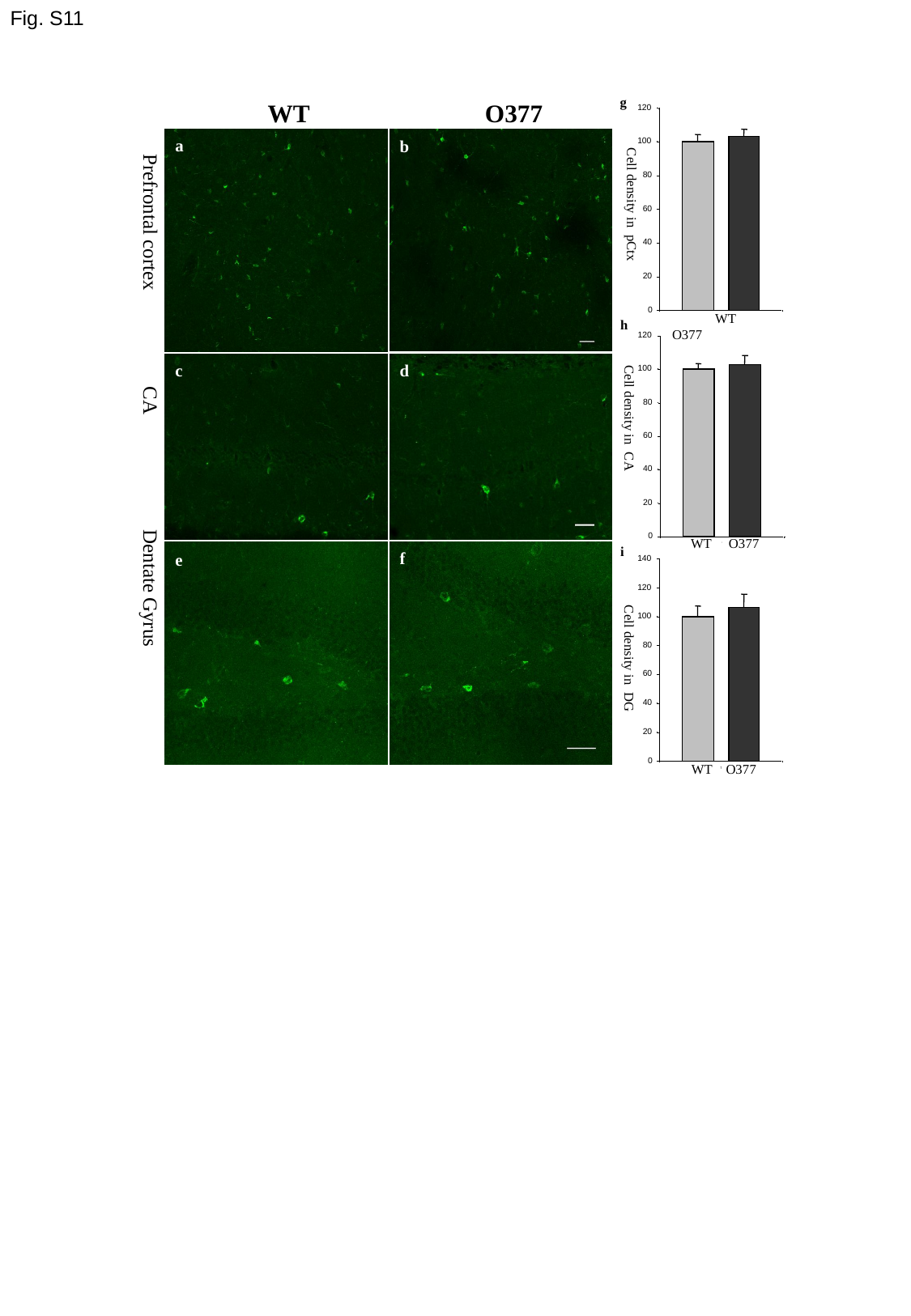

Fig. S11
g
 WT O377
120
 Prefrontal cortex CA Dentate Gyrus
a
b
100
 Cell density in pCtx
80
60
40
20
0
 WT O377
h
1
120
c
d
 Cell density in CA
100
80
60
40
20
 WT O377
0
i
1
f
e
140
120
Cell density in DG
100
80
60
40
20
0
 WT O377
1

## Slide 13
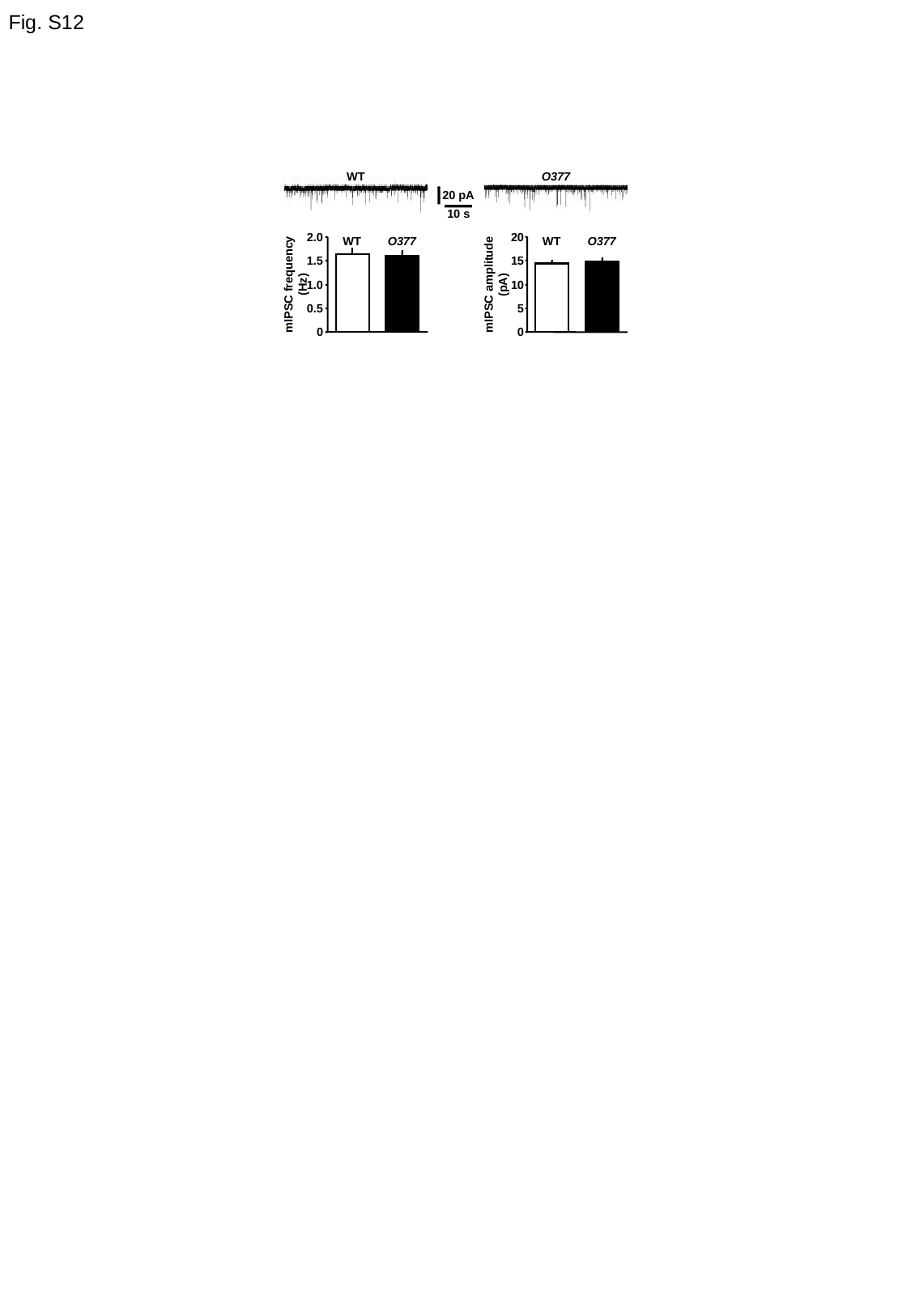

Fig. S12
WT
O377
20 pA
10 s
WT
O377
WT
O377
2.0
20
1.5
15
mIPSC frequency
(Hz)
mIPSC amplitude
(pA)
1.0
10
0.5
5
0
0
